# Low‐intensity shockwave therapy for erectile dysfunction: An abridged Cochrane review

**DOI:** 10.1111/bju.70236

**Published:** 2026-03-30

**Authors:** Onuralp Ergun, Kwangmin Kim, Myung Ha Kim, Eu Chang Hwang, Yooni Blair, Ahmet Gudeloglu, Sijo Parekattil, Philipp Dahm

**Affiliations:** ^1^ Department of Urology University of Minnesota Minneapolis MN USA; ^2^ Urology Section Minneapolis VA Health Care System Minneapolis MN USA; ^3^ Surgery Yonsei University Wonju College of Medicine Wonju South Korea; ^4^ Yonsei Wonju Medical Library Yonsei University Wonju College of Medicine Wonju South Korea; ^5^ Department of Urology Chonnam National University Medical School, Chonnam National University Hwasun Hospital Hwasun South Korea; ^6^ Department of Urology University of Michigan Ann Arbor MI USA; ^7^ Department of Urology Hacettepe University Ankara Turkey; ^8^ Avant Concierge Urology Winter Garden FL USA

**Keywords:** erectile dysfunction, extracorporeal shockwave therapy, patient satisfaction, quality of life, randomised controlled trials, treatment outcome

## Abstract

**Objective:**

To assess the efficacy and safety of low‐intensity shockwave therapy (LiSWT) for treating erectile dysfunction (ED) compared to sham therapy.

**Methods:**

This summary is based on the Cochrane systematic review published in the *Cochrane Database of Systematic Reviews* 2025, Issue 7 (CD013166). We included randomised controlled trials (RCTs) comparing LiSWT to sham therapy. We excluded psychogenic or iatrogenic ED, kidney transplants, or radical prostatectomy. Critical outcomes were erectile function, discontinuation, and adverse events; important outcomes included rigidity, satisfaction, and quality of life. Certainty was assessed using the Grading of Recommendations, Assessment, Development, and Evaluation (GRADE) approach.

**Results:**

A total of 21 RCTs (1357 men, aged 39–65 years) were analysed. LiSWT may slightly improve short‐term erectile function (mean difference [MD] 3.89, 95% confidence interval [CI] 2.89–4.89; *I* ^2^ = 62%; 15 studies; low‐certainty), though clinical importance is uncertain. Long‐term function may improve (MD 5.25, 95% CI 2.47–8.04; *I* ^2^ = 87%; five studies; low‐certainty). LiSWT likely has little effect on discontinuation (risk ratio 0.77, 95% CI 0.47–1.27; *I* ^2^ = 0%) or adverse events (risk difference 0.00, 95% CI −0.01 to 0.02; *I* ^2^ = 0%). It may improve short‐term penile rigidity (MD 1.06, 95% CI 0.83–1.28; *I* ^2^ = 53%; low‐certainty). Data for satisfaction or quality of life were unavailable.

**Conclusions:**

Low‐intensity shockwave therapy may improve long‐term erectile function and short‐term rigidity, with little difference in discontinuation or adverse events. Evidence certainty is low due to methodological limitations and heterogeneity.

**Abbreviations**
ED: erectile dysfunction
EHS: Erection Hardness Score
GRADE: Grading of Recommendations, Assessment, Development, and Evaluation (approach)
IIEF‐EF: International Index of Erectile Function‐Erectile Function domain
LiSWT: low‐intensity shockwave therapy
MCID: minimal clinically important difference
MD: mean difference
PRISMA: Preferred Reporting Items for Systematic Reviews and Meta‐Analyses
RCT: randomised controlled trial
RD: risk difference
RR: risk ratio

## Introduction

Erectile dysfunction (ED) is defined as the consistent or recurrent inability to attain and/or maintain, a penile erection sufficient for sexual satisfaction, satisfactory performance, or both [1]. ED is a common condition affecting men of all ages, with a prevalence of ~40% in men aged 40–49 years and up to 90% in those >70 years [1]. Low‐intensity shockwave therapy (LiSWT) has recently emerged as a potential restorative treatment option for ED, aiming to enhance penile haemodynamics through neovascularisation and tissue regeneration [25, 26]. Several studies have examined its use, but their findings remain inconsistent, and the clinical significance of observed improvements is uncertain.

## Methods

This systematic review and meta‐analysis was based on a published protocol [42]. Our research question was structured according to the PICO (population, intervention, comparator, outcome) format. We applied a rigorous methodology to assess LiSWT, with a focus on patient‐important outcomes, a comprehensive literature search, and the use of the Grading of Recommendations, Assessment, Development, and Evaluation (GRADE) approach reported in the summary of findings tables (Tables 1 and 2). Our detailed search strategy is presented in Appendix S1.

**Table 1:** Summary of findings (short term, ≤3 months).

LiSWT for ED (short term)						
Patient or population: men aged ≥18 years with organic or mixed ED						
Setting: outpatient setting						
Intervention: LiSWT						
Comparison: sham therapy						
Outcomes*	Anticipated absolute effects** (95% CI)		Relative effect (95% CI)	No. of participants (studies)	Certainty of the evidence (GRADE)***	Interpretation
	Risk with sham therapy	Risk difference with LiSWT				
**Erectile function** Assumed MCID: 4 point change in IIEF‐EF score (6–30 scale; higher means better)	Mean IIEF‐EF score ranged from 8.6 to 20.9	**MD 3.89 points higher** (2.89 higher to 4.89 higher)	—	937 (15 RCTs)	⊕⊕⊝⊝ Low^†^ ^,^ ^‡^	There may be a small effect on erectile function. However, based on the selected MCID, this small effect may not be clinically important
**Discontinuation from treatment** Assumed MCID: 5% absolute change	64 per 1000	**15 fewer per 1000** (34 fewer to 17 more)	**RR 0.77** (0.47–1.27)	1132 (17 RCTs)	⊕⊕⊝⊝ Low^†^ ^,^ ^§^	LiSWT may have little to no effect on discontinuation from treatment between LiSWT and sham therapy
**Treatment‐related adverse events** Assumed MCID: 5% absolute change	7 per 1000	**RD 0.0** (−0.01 to 0.02)		1400 (20 RCTs)	⊕⊕⊝⊝ Low^†^ ^,^ ^¶^	LiSWT may have little to no effect on treatment‐related adverse events between LiSWT and sham therapy
**Patient/partner satisfaction**	—	—	—	—	—	We found no evidence on how LiSWT may affect patient/partner satisfaction
**Penile rigidity** Assumed MCID: 1 point change in EHS score (1–4 scale; higher means better)	The mean EHS score ranged from 1.4 to 2.4.	**MD 1.06 points higher** (0.83 higher to 1.28 higher)	—	252 (4 RCTs)	⊕⊕⊝⊝ Low^†^ ^,^ ^§^	LiSWT may improve penile rigidity compared to sham therapy
**Sexual quality of life**	—	—	—	—	—	We found no evidence on how LiSWT may affect sexual quality of life

*Note:* Bold values represent the risk difference and the relative effects followed by the confidence interval in parenthesis.

Abbreviations: MD, mean difference; RR, relative risk; RD, risk difference.

* The risk in the intervention group (and its 95% CI) is based on the assumed risk in the comparison group and the relative effect of the intervention (and its 95% CI).

** Analysed using random‐effects models because of heterogeneity. Follow‐up duration is calculated as a weighted average of studies in each meta‐analysis.

*** GRADE Working Group grades of evidence. High certainty: we are very confident that the true effect lies close to that of the estimate of the effect. Moderate certainty: we are moderately confident in the effect estimate; the true effect is likely to be close to the estimate of the effect, but there is a possibility that it is substantially different. Low certainty: our confidence in the effect estimate is limited; the true effect may be substantially different from the estimate of the effect. Very low certainty: we have very little confidence in the effect estimate; the true effect is likely to be substantially different from the estimate of effect.

^†^ Downgraded by one level for study limitations: unclear or high risk of bias in half or more domains.

^‡^ Downgraded by one level for clinically important, serious inconsistency. Imprecision caused by a wide CI crossing the assumed MCID threshold contributed to this decision to rate down once.

^§^ Downgraded by one level for clinically important, serious imprecision: wide CI crossing the MCID threshold once.

^¶^ Downgraded by one level for clinically important, serious imprecision: wide CI crossing the null value once.

**Table 2:** Summary of findings (long term, >3 months).

LiSWT for ED (long term)						
Patient or population: men aged ≥18 years with organic or mixed ED						
Setting: outpatient setting						
Intervention: LiSWT						
Comparison: sham therapy						
Outcomes*	Anticipated absolute effects** (95% CI)		Relative effect (95% CI)	No. of participants (studies)	Certainty of the evidence (GRADE)***	Interpretation
	Risk with sham therapy	Risk difference with LiSWT				
**Erectile function** Assumed MCID: 4‐point change in IIEF‐EF score (6–30 scale; higher means better)	Mean IIEF‐EF scores ranged from 8 to 19.8	**MD 5.25 points higher** (2.47 higher to 8.04 higher)	—	276 (5 RCTs)	⊕⊕⊝⊝ Low^†^ ^,^ ^‡^	LiSWT may improve erectile function compared to sham therapy
**Discontinuation from treatment** Assumed MCID: 5% absolute change	—	—	—	—	—	We found no evidence on how LiSWT may affect discontinuation from treatment^§^
**Treatment‐related adverse events** Assumed MCID: 5% absolute change	0 per 1000	**RD 0.0** (−0.02 to 0.02)		411 (6 RCTs)	⊕⊕⊝⊝ Low^†^ ^,^ ^¶^	LiSWT may have little to no effect on treatment‐related adverse events between LiSWT and sham therapy
**Patient/partner satisfaction**	—	—	—	—	—	We found no evidence on how LiSWT may affect patient/partner satisfaction
**Penile rigidity** Assumed MCID: 1 point change in EHS score (1–4 scale; higher means better)	Mean EHS score ranged from 1.3 to 2.4.	**MD 0.91 points higher** (0.36 higher to 1.46 higher)	—	169 (3 RCTs)	⊕⊕⊝⊝ Low^†^ ^,^ ^‡^	LiSWT may have a small effect on penile rigidity. However, based on the selected MCID, this small effect may not be clinically important
**Sexual quality of life**	—	—	—	—	—	We found no evidence on how LiSWT may affect sexual quality of life

*Note:* Bold values represent the risk difference and the relative effects followed by the confidence interval in parenthesis.

Abbreviations: MD, mean difference; RR, relative risk; RD, risk difference.

* The risk in the intervention group (and its 95% CI) is based on the assumed risk in the comparison group and the relative effect of the intervention (and its 95% CI).

** Analysed using random‐effects models because of heterogeneity. Follow‐up duration is calculated as a weighted average of studies in each meta‐analysis.

*** GRADE Working Group grades of evidence. High certainty: we are very confident that the true effect lies close to that of the estimate of the effect. Moderate certainty: we are moderately confident in the effect estimate; the true effect is likely to be close to the estimate of the effect, but there is a possibility that it is substantially different. Low certainty: our confidence in the effect estimate is limited; the true effect may be substantially different from the estimate of the effect. Very low certainty: we have very little confidence in the effect estimate; the true effect is likely to be substantially different from the estimate of effect.

^†^ Downgraded by one level for study limitations: unclear or high risk of bias in half or more domains.

^‡^ Downgraded by one level for clinically important, serious inconsistency. Imprecision caused by a wide CI crossing the assumed MCID threshold contributed to this decision to rate down once.

^§^ There were no studies with a treatment period >3 months. Therefore, we also found no data for this outcome.

^¶^ Downgraded by one level for clinically important, serious imprecision: wide CI crossing the null value once.

**Fig. 1:**
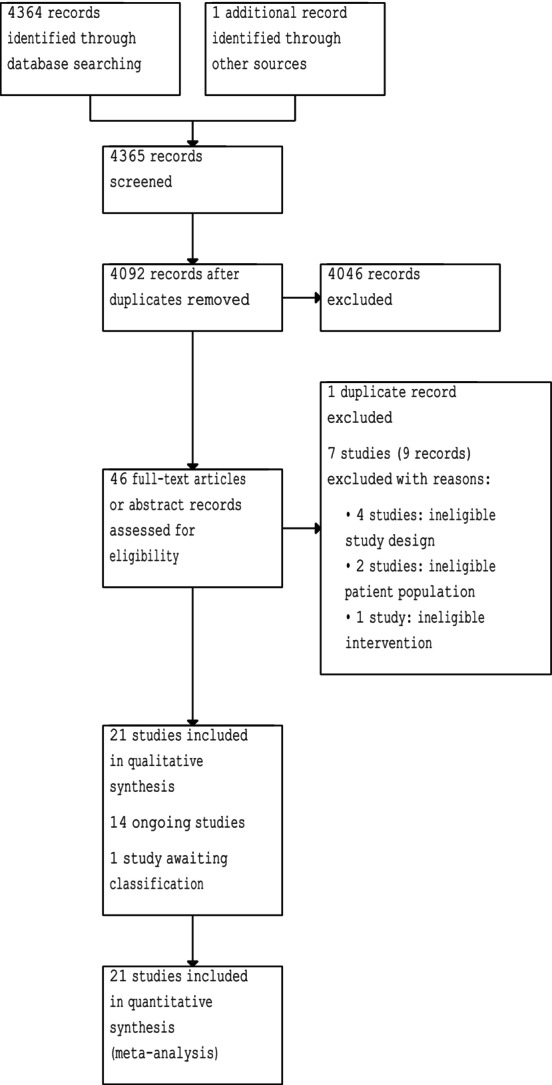
The PRISMA flow diagram.

Two review authors (O.E., K.K.) independently scanned the abstract or title, or both, of the remaining records retrieved, to determine which studies we should assess further. Working independently, two review authors (O.E., K.K.) then investigated all potentially relevant records in full text, mapped records to studies, and classified studies as included studies (Table S1). We resolved any discrepancies through consensus or recourse to a third review author (P.D.). The Preferred Reporting Items for Systematic Reviews and Meta‐Analyses (PRISMA) flowchart illustrates this process (Fig. 1). Searches were conducted up to 7 July 2024 without language or publication restrictions.

We included randomised controlled trials (RCTs) comparing LiSWT with sham therapy in men aged ≥18 years with organic or mixed ED. Trials using electrohydraulic, electromagnetic, or piezoelectric LiSWT generators were eligible. Trials involving men with iatrogenic, psychogenic, or neurological causes of ED were excluded (Table S2). Included studies are reported in Table S1. Critical outcomes included erectile function, discontinuation from treatment, and treatment‐related adverse events. Important outcomes included penile rigidity, patient/partner satisfaction, and sexual quality of life. Data were synthesised using random‐effects meta‐analysis, and the certainty of evidence was rated using the GRADE approach [70].

**Fig. 2:**
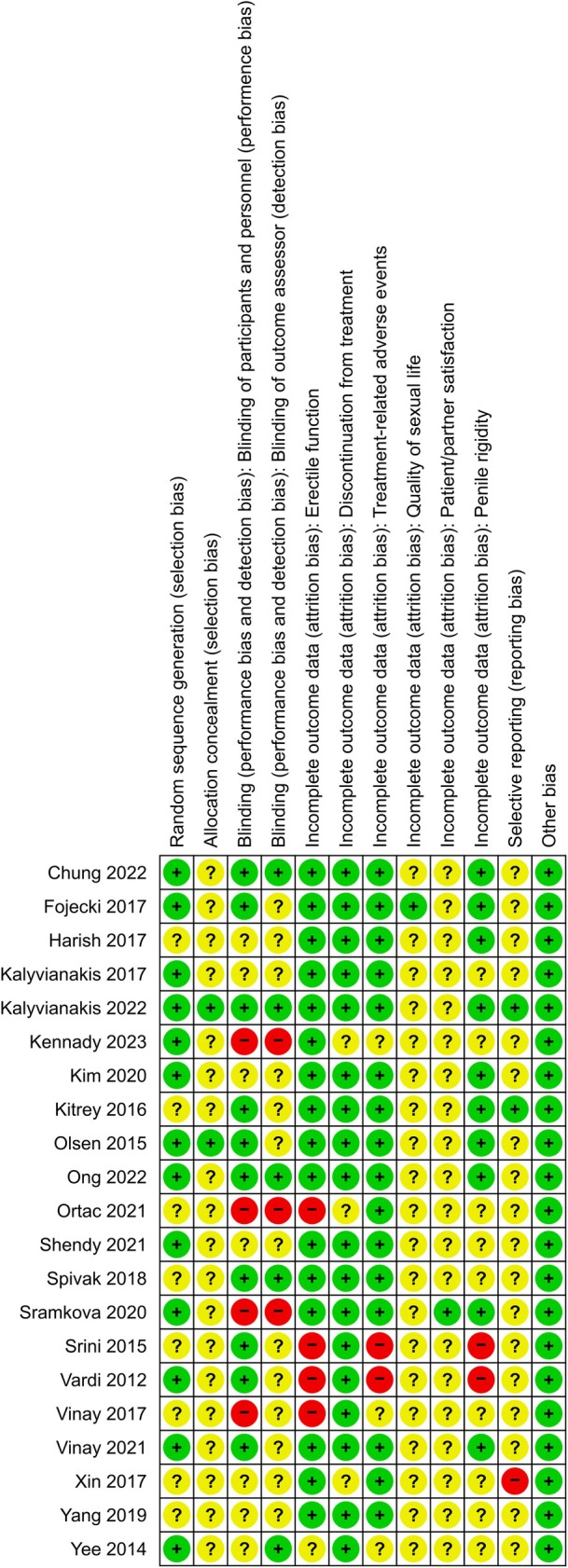
Risk of bias summary: the review authors’ judgements about each risk of bias item for each included study.

**Fig. 3:**
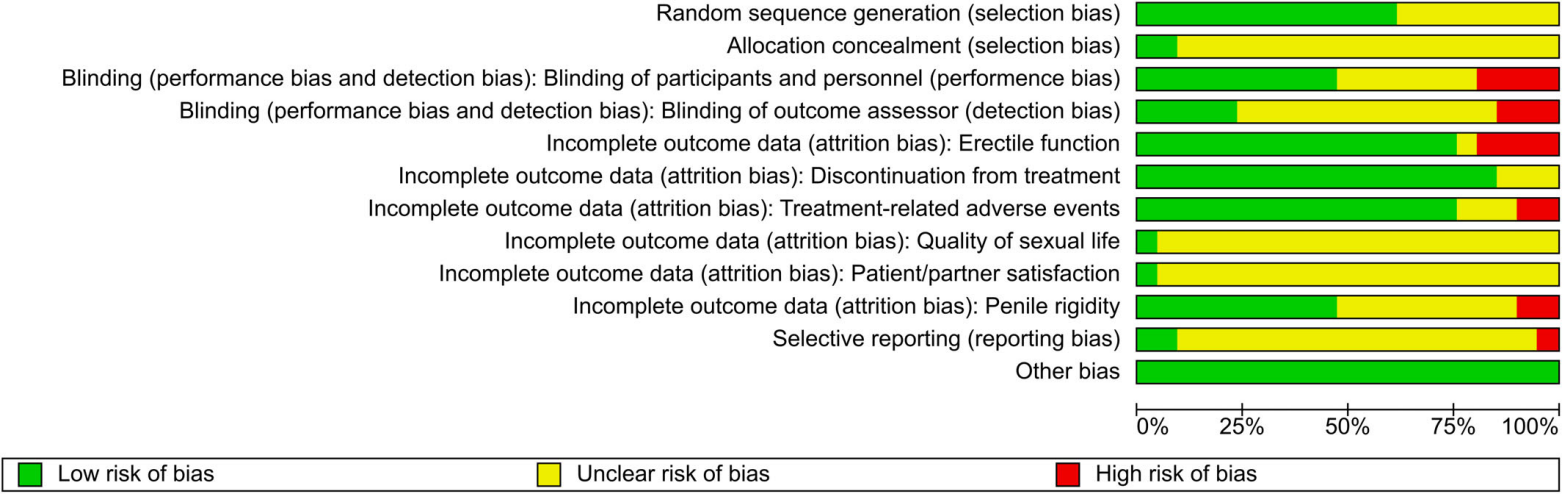
Risk of bias summary with CIs: the review authors’ judgements about each risk of bias item for each included study.

Two review authors (O.E., K.K.) independently assessed the risk of bias in each included study. We resolved disagreements again, by consensus, or by consulting a third review author (P.D.). We assessed risk of bias using Cochrane's risk of bias assessment tool [60] (Figs 2 and 3).

We included the domains random sequence generation (selection bias), allocation concealment (selection bias), blinding of participants and personnel (performance bias), blinding of outcome assessment (detection bias), incomplete outcome data (attrition bias), selective reporting (reporting bias), and other sources of bias. The certainty of evidence was rated independently by two review authors (O.E., P.D.) on a per‐outcome basis using the GRADE approach.

For concise reporting purposes, this article summarises the key findings of the Cochrane review (Ergun et al., *Cochrane Database of Systematic Reviews* 2025, Issue 7, CD013166), focusing on the primary outcomes of clinical importance. Full methodological details, sensitivity analyses, subgroup analyses, and extended outcome data can be found in the published Cochrane version [42].

## Results

### Literature Search Results

A total of 21 RCTs (1357 participants) were included. Details of the search are provided in the PRISMA flow diagram (Fig. 1). Characteristics of included studies are shown in Table S1. A summary of the risk of bias of these studies is presented in Figs 1 and 2.

### Participants

The mean age of participants ranged between 39.8 and 65.1 years. Baseline mean International Index of Erectile Function‐Erectile Function (IIEF‐EF) domain scores ranged from 7 to 20. Treatment protocols differed across studies: active intervention duration ranged from 2 to 9 weeks, and most studies established a wash‐out period for the use of phosphodiesterase type 5 inhibitors before the first LiSWT session. Treatment frequency, number of shocks, energy density, and frequency settings varied in each study and are demonstrated in the overview of included studies table (Table 1).

### Effects of Interventions

All results are presented in the ‘Summary of findings’ tables (Tables 1 and 2).

#### Short‐Term Outcomes (≤3 months)

Erectile function: LiSWT may have a small effect on erectile function in the short term (mean difference [MD] 3.89 points higher on the IIEF‐EF scale, 95% CI 2.89–4.89; *I* ^2^ = 62%; 15 studies, 937 participants; low‐certainty evidence, although this may not be clinically important; Table 1).

Discontinuation from treatment: LiSWT may have little to no effect on discontinuation rates (risk ratio [RR] 0.77, 95% CI 0.47–1.27; *I* ^2^ = 0%; 17 studies, 1132 participants; Table 1).

Treatment‐related adverse events: LiSWT may have little to no effect on adverse events (risk difference [RD] 0.00, 95% CI −0.01 to 0.02; *I* ^2^ = 0%; 20 studies, 1400 participants; Table 1).

Penile rigidity: LiSWT may improve penile rigidity (MD 1.06 points higher on the Erection Hardness Score [EHS], 95% CI 0.83–1.28; *I* ^2^ = 53%; four studies, 252 participants; low‐certainty evidence; Table 1).

Patient/partner satisfaction and sexual quality of life: No data were available.

#### Long‐Term Outcomes (>3 months)

Erectile function: LiSWT may improve erectile function in the long term (MD 5.25 points higher on the IIEF‐EF scale, 95% CI 2.47–8.04; *I* ^2^ = 87%; five studies, 276 participants; low‐certainty evidence; Table 2).

Discontinuation from treatment: no data were available for the long term.

Treatment‐related adverse events: LiSWT may have little to no effect on adverse events in the long term (RD 0.00, 95% CI −0.02 to 0.02; *I* ^2^ = 0%; six studies, 411 participants; Table 2).

Penile rigidity: LiSWT may have a small positive effect in the long term (MD 0.91 points higher on the EHS, 95% CI 0.36–1.46; *I* ^2^ = 89%; three studies, 169 participants; low‐certainty evidence, although this may not be clinically important; Table 2).

Patient/partner satisfaction and sexual quality of life: No data were available.

The certainty of evidence for all reported outcomes was low, primarily due to inconsistency, imprecision, and study limitations. Summary of these findings and certainty of evidence assessments are illustrated in the summary of findings tables (Tables 1 and 2).

## Discussion

In this systematic review and meta‐analysis, we present the most up‐to‐date and comprehensive evidence summary currently available on the topic of LiSWT.

Low‐intensity shockwave therapy may provide modest improvements in erectile function and penile rigidity, with minimal associated risks. However, the clinical importance of these improvements remains uncertain given that the observed mean differences in IIEF‐EF and EHS scores are close to or below established minimal clinically important differences (MCIDs).

We downgraded the certainty of evidence for all outcomes consistently by two levels (low‐certainty evidence). Our reasons for downgrading the evidence are categorised below.

- Study limitations: almost all the included studies failed to describe the method of allocation concealment. More than half of the studies had issues with the blinding of study participants, personnel, and outcome assessors. There was a high risk of attrition bias in a number of studies. Only two studies were conducted according to a pre‐registered protocol. Most studies did not provide details on a protocol, and one study had a registered protocol that the study authors deviated from and selectively reported their outcomes.
- Inconsistency: corresponded to a high *I* ^2^ value. Our pre‐defined secondary analyses (subgroup and sensitivity analyses) failed to explain the observed heterogeneity. Aside from chance variation and methodological variety, clinical differences in each study participant profile (age, ED duration, baseline ED severity, ED aetiology, etc.) are the potential source of these observed differences between individual study results.
- Imprecision: the decision to downgrade due to imprecision was made when the CIs around the point estimate crossed pre‐defined MCID thresholds. In some instances, inconsistency appeared to be the underlying reason for the apparent imprecision. On such occasions, we did not downgrade further.
- Only a few studies reported long‐term results, and these were studies with more favourable short‐term results. This raises concerns around potential bias with longer‐term results being reported selectively by those studies with ‘positive’ outcomes. The lack of protocols for most included studies prohibited us from investigating this issue further.

We used a partially contextualised approach to investigate the results of this review beyond the concept of statistical significance by contemplating thresholds for MCIDs. Our MCIDs were informed by the literature and the clinical judgement of our content experts and were defined a priori. We recognise that choosing different thresholds would have affected the interpretation of the results. Specifically, Rosen et al. [49] have proposed IIEF‐EF thresholds of 2, 5, and 7 for men with mild, moderate, and severe ED, respectively. As we expected most studies to include mixed populations of men with mild to moderate ED severity, we planned for an MCID of 4 as captured in our published protocol [42]. As different assumptions about thresholds may result in different judgements about the effect sizes, we sought to make our judgements as transparent as possible to the readers.

## Conclusion

Low‐intensity shockwave therapy may improve erectile function in the long term and penile rigidity in the short term, with little to no difference in discontinuation or adverse events compared to sham therapy. However, the certainty of evidence remains low due to methodological limitations and heterogeneity among studies. The clinical importance of the observed improvements is uncertain. Larger, well‐designed RCTs with standardised protocols and patient‐important outcomes are needed to confirm these findings and define the role of LiSWT in the management of erectile dysfunction.

## Disclosure of Interests

Onuralp Ergun: was a Fellow of Cochrane Urology; however, he was not involved in the editorial process of this review. Kwangmin Kim: no conflicts exist. Myung Ha Kim: no conflicts exist. Eu Chang Hwang: is a Contact Editor for Cochrane Urology; however, he was not involved in the editorial process of this review. Yooni Blair: no conflicts exist. Ahmet Gudeloglu: no conflicts exist. Sijo Parekattil: no conflicts exist. Philipp Dahm: is the Co‐ordinating Editor of Cochrane Urology; however, he was not involved in the editorial process of this review.

## Supporting information


**Appendix S1.** Search strategies.

**Table S1.** Characteristics of included studies.

**Table S2.** Characteristics of excluded studies.
