# Supplementary material for: Low‐intensity shockwave therapy for erectile dysfunction: An abridged Cochrane review
Source: BJU Int. 2026 Mar 30;137(6):949–57. doi: 10.1111/bju.70236 (PMC13168926; doi:10.1111/bju.70236)
Supplement: Supplementary file 1 — Appendix S1. Search strategies. [file BJU-137-949-s002.docx]

**Supplement 1. Search strategies**

**Ergun O, Kim K, Kim MH, Hwang EC, Blair Y, Gudeloglu A, Parekattil S, Dahm P**
<https://doi.org/10.1002/14651858.CD013166.pub3>

*The material in this section has been supplied by the author(s) for publication under a Licence for Publication and the author(s) are solely responsible for the material. Cochrane has peer reviewed this material in accordance with its*[*editorial policies*](https://www.cochranelibrary.com/cdsr/editorial-policies)*, but Cochrane has not copyedited, formatted or proofread. Cochrane accordingly gives no representations or warranties of any kind in relation to, and accepts no liability for any reliance on or use of, such material.*

**Search strategies**

| **1.1. MEDLINE via Ovid (from 1946)**[**https://ovidsp.ovid.com/**](https://ovidsp.ovid.com/) | |
| --- | --- |
| #1 | exp Extracorporeal Shockwave Therapy/ |
| #2 | exp Ultrasonic Therapy/ |
| #3 | High-Energy Shock Waves/ |
| #4 | (shock wave* or shockwave*).tw. |
| #5 | (Ultraso* adj2 therap*).tw. |
| #6 | ESWT.tw. |
| #7 | ECSW.tw. |
| #8 | #1 or #2 or #3 or #4 or #5 or #6 or #7 |
| #9 | exp Erectile Dysfunction/ |
| #10 | Erectile dysfunction*.tw. |
| #11 | Erectile function*.tw. |
| #12 | Erectile failure*.tw. |
| #13 | Erection failure*.tw. |
| #14 | ED.tw. |
| #15 | Penile erect*.tw. |
| #16 | Impoten*.tw. |
| #17 | (sexual adj (function* or dysfunction* or problem* or symptom* or arous*)).tw. |
| #18 | (sex adj (function* or dysfunction* or problem* or symptom* or arous*)).tw. |
| #19 | exp Impotence, Vasculogenic/ |
| #20 | #9 or #10 or #11 or #12 or #13 or #14 or #15 or #16 or #17 or #18 or #19 |
| #21 | #8 and #20 |
| #22 | randomized controlled trial.pt. |
| #23 | controlled clinical trial.pt. |
| #24 | randomized.ab. |
| #25 | placebo.ab. |
| #26 | drug therapy.fs. |
| #27 | randomly.ab. |
| #28 | trial.ab. |
| #29 | groups.ab. |
| #30 | #22 or #23 or #24 or #25 or #26 or #27 or #28 or #29 |
| #31 | exp animals/ not humans.sh. |
| #32 | #30 not #31 |
| #33 | #21 and #32 |
| **1.2. Embase via Elsevier (from 1974) https://www.embase.com/** | |
| #1 | 'shock wave therapy'/exp |
| #2 | 'ultrasound therapy'/exp |
| #3 | 'high-energy shock wave'/de |
| #4 | 'shock wave*':ti,ab OR shockwave*:ti,ab |
| #5 | (ultraso* NEAR/2 therap*):ti,ab |
| #6 | eswt:ti,ab |
| #7 | ecsw:ti,ab |
| #8 | #1 OR #2 OR #3 OR #4 OR #5 OR #6 OR #7 |
| #9 | 'erectile dysfunction'/exp |
| #10 | 'erectile dysfunction*':ti,ab |
| #11 | 'erectile function*':ti,ab |
| #12 | 'erectile failure*':ti,ab |
| #13 | 'erection failure*':ti,ab |
| #14 | ed:ti,ab |
| #15 | 'penile erect*':ti,ab |
| #16 | impoten*:ti,ab |
| #17 | (sexual NEXT/1 (function* OR dysfunction* OR problem* OR symptom* OR arous*)):ti,ab |
| #18 | (sex NEXT/1 (function* OR dysfunction* OR problem* OR symptom* OR arous*)):ti,ab |
| #19 | 'impotence'/exp |
| #20 | #9 OR #10 OR #11 OR #12 OR #13 OR #14 OR #15 OR #16 OR #17 OR #18 OR #19 |
| #21 | #8 AND #20 |
| #22 | (random*:ti,ab,tt OR 'randomization'/de OR 'intermethod comparison'/de OR placebo:ti,ab,tt OR compare:ti,tt OR compared:ti,tt OR comparison:ti,tt OR ((evaluated:ab OR evaluate:ab OR evaluating:ab OR assessed:ab OR assess:ab) AND (compare:ab OR compared:ab OR comparing:ab OR comparison:ab)) OR ((open NEXT/1 label):ti,ab,tt) OR (((double OR single OR doubly OR singly) NEXT/1 (blind OR blinded OR blindly)):ti,ab,tt) OR 'double blind procedure'/de OR ((parallel NEXT/1 group*):ti,ab,tt) OR crossover:ti,ab,tt OR 'cross over':ti,ab,tt OR (((assign* OR match OR matched OR allocation) NEAR/6 (alternate OR group OR groups OR intervention OR interventions OR patient OR patients OR subject OR subjects OR participant OR participants)):ti,ab,tt) OR assigned:ti,ab,tt OR allocated:ti,ab,tt OR ((controlled NEAR/8 (study OR design OR trial)):ti,ab,tt) OR volunteer:ti,ab,tt OR volunteers:ti,ab,tt OR 'human experiment'/de OR trial:ti,tt) NOT ('randomized controlled trial'/de OR 'controlled clinical trial'/de) NOT (((random* NEXT/1 sampl* NEAR/8 ('cross section*' OR questionnaire* OR survey OR surveys OR database OR databases)):ti,ab,tt) NOT ('comparative study'/de OR 'controlled study'/de OR 'randomised controlled':ti,ab,tt OR 'randomized controlled':ti,ab,tt OR 'randomly assigned':ti,ab,tt) OR ('cross-sectional study'/de NOT ('randomized controlled trial'/de OR 'controlled clinical study'/de OR 'controlled study'/de OR 'randomised controlled':ti,ab,tt OR 'randomized controlled':ti,ab,tt OR 'control group':ti,ab,tt OR 'control groups':ti,ab,tt)) OR ('case control*':ti,ab,tt AND random*:ti,ab,tt NOT ('randomised controlled':ti,ab,tt OR 'randomized controlled':ti,ab,tt)) OR ('systematic review':ti,tt NOT (trial:ti,tt OR study:ti,tt)) OR (nonrandom*:ti,ab,tt NOT random*:ti,ab,tt) OR 'random field*':ti,ab,tt OR (('random cluster' NEAR/4 sampl*):ti,ab,tt) OR (review:ab AND review:it NOT trial:ti,tt) OR ('we searched':ab AND (review:ti,tt OR review:it)) OR 'update review':ab OR ((databases NEAR/5 searched):ab) OR ((rat:ti,tt OR rats:ti,tt OR mouse:ti,tt OR mice:ti,tt OR swine:ti,tt OR porcine:ti,tt OR murine:ti,tt OR sheep:ti,tt OR lambs:ti,tt OR pigs:ti,tt OR piglets:ti,tt OR rabbit:ti,tt OR rabbits:ti,tt OR cat:ti,tt OR cats:ti,tt OR dog:ti,tt OR dogs:ti,tt OR cattle:ti,tt OR bovine:ti,tt OR monkey:ti,tt OR monkeys:ti,tt OR trout:ti,tt OR marmoset*:ti,tt) AND 'animal experiment'/de) OR ('animal experiment'/de NOT ('human experiment'/de OR 'human'/de))) |
| #23 | #21 AND #22 |
| **1.3. Cochrane Library (https://www.cochranelibrary.com/)** | |
| #1 | [mh "Extracorporeal Shockwave Therapy"] |
| #2 | [mh "Ultrasonic Therapy"] |
| #3 | [mh ^"High-Energy Shock Waves"] |
| #4 | ("shock wave*" or shockwave*):ti,ab,kw |
| #5 | (ultraso* near/2 therap*):ti,ab,kw |
| #6 | eswt:ti,ab,kw |
| #7 | ecsw:ti,ab,kw |
| #8 | #1 or #2 or #3 or #4 or #5 or #6 or #7 |
| #9 | [mh "Erectile Dysfunction"] |
| #10 | ((Erectile or erection) near/1 (dysfunction* or function* or failure*)):ti,ab,kw |
| #11 | ED:ti,ab,kw |
| #12 | ("Penile erect*"):ti,ab,kw |
| #13 | Impoten*:ti,ab,kw |
| #14 | (sexual next/1 (function* OR dysfunction* OR problem* OR symptom* OR arous*)):ti,ab,kw |
| #15 | (sex next/1 (function* OR dysfunction* OR problem* OR symptom* OR arous*)):ti,ab,kw |
| #16 | [mh "Impotence, Vasculogenic"] |
| #17 | #9 or #10 or #11 or #12 or #13 or #14 or #15 or #16 |
| #18 | #8 and #17 |
| **1.4. Scopus (https://www.scopus.com/)** | |
| #1 | TITLE-ABS("shock wave*" OR shockwave* OR (Ultraso* W/2 therap*) OR ESWT OR ECSW) AND TITLE-ABS(((Erectile or erection) Pre/1 (dysfunction* or function* or failure*)) OR (Penile Pre/1 erect*) OR Impoten* OR (sex* Pre/1 (function* OR dysfunction* OR problem* OR symptom* OR arous*))) AND ( INDEXTERMS ( "clinical trials" OR "clinical trials as a topic" OR "randomized controlled trial" OR "Randomized Controlled Trials as Topic" OR "controlled clinical trial" OR "Controlled Clinical Trials" OR "random allocation" OR "Double-Blind Method" OR "Single-Blind Method" OR "Cross-Over Studies" OR "Placebos" OR "multicenter study" OR "double blind procedure" OR "single blind procedure" OR "crossover procedure" OR "clinical trial" OR "controlled study" OR "randomization" OR "placebo" ) ) OR ( TITLE-ABS-KEY ( ( "clinical trials" OR "clinical trials as a topic" OR "randomized controlled trial" OR "Randomized Controlled Trials as Topic" OR "controlled clinical trial" OR "Controlled Clinical Trials as Topic" OR "random allocation" OR "randomly allocated" OR "allocated randomly" OR "Double-Blind Method" OR "Single-Blind Method" OR "Cross-Over Studies" OR "Placebos" OR "cross-over trial" OR "single blind" OR "double blind" OR "factorial design" OR "factorial trial" ) ) ) OR ( TITLE-ABS ( clinical trial* OR trial* OR rct* OR random* OR blind* ) ) |
| **1.5. Web of Science (https://www.webofscience.com/)** | |
| #1 | TS=("shock wave*" OR shockwave* OR (Ultraso* NEAR/2 Therap*) OR ESWT OR ECSW) AND TS=(((Erectile or erection) NEAR/1 (dysfunction* or function* or failure*)) OR (Penile NEAR/1 erect*) OR Impoten* OR (sex* NEAR/1 (function* OR dysfunction* OR problem* OR symptom* OR arous*))) AND (TS=(randomised OR randomized OR randomisation OR randomisation OR placebo* OR (random* AND (allocat* OR assign*)) OR (blind* AND (single OR double OR treble OR triple)))) |
| **1.6** | **Latin American and Caribbean Health Sciences Literature (https://lilacs.bvsalud.org/en/)** |
|  | (mh:("Erectile Dysfunction" OR "Impotence, Vasculogenic" OR "High-Energy Shock Waves") OR tw:(((erectile OR erection) AND (dysfunction* or function* or failure*)) OR impoten$ OR "penile erection" OR ((sex OR sexual) AND (function* OR dysfunction* OR problem* OR symptom* OR arous*)))) AND ((mh:("Extracorporeal Shockwave Therapy" OR "Ultrasonic Therapy")) OR (tw:("shock wave*" OR shockwave* OR ultraso* OR eswt OR ecsw))) AND ((PT:"randomized controlled trial" OR PT:"controlled clinical trial" OR PT:"multicenter study" OR MH:"randomized controlled trials as topic" OR MH:"controlled clinical trials as topic" OR MH:"multicenter studies as topic" OR MH:"random allocation" OR MH:"double-blind method" OR MH:"single-blind method") OR ((ensaio$ OR ensayo$ OR trial$) AND (azar OR acaso OR placebo OR control$ OR aleat$ OR random$ OR enmascarado$ OR simpleciego OR ((simple$ OR single OR duplo$ OR doble$ OR double$) AND (cego OR ciego OR blind OR mask))) AND clinic$)) AND NOT (MH:animals OR MH:rabbits OR MH:rats OR MH:primates OR MH:dogs OR MH:cats OR MH:swine OR PT:"in vitro") |
| **1.7. ClinicalTrials.gov (https://clinicaltrials.gov/)** | |
| #2 | Condition or disease = (erectile OR impotence OR impotent OR "penile erection" OR "sexual dysfunction" OR "sexual function" OR "sexual problem" OR "sexual symptom" OR "sexual arousal") |
| #2 | Other terms = ("shock wave" OR shockwave OR ESWT OR ECSW) |
| #3 | 1 AND 2 |
| **1.8. World Health Organization International Clinical Trials Registry Platform search portal (http://apps.who.int/trialsearch/)** | |
| #1 | Basic search = (erectile OR impotence OR impotent OR erection) AND (shock wave OR shockwave OR ESWT OR ECSW) |
| **1.9. Grey Literature Report (https://catalog.nyam.org/)** | |
| #1 | (erectile OR erection OR impotence OR impotent) AND (shockwave OR shock wave) |
