# Supplementary material for: Low‐intensity shockwave therapy for erectile dysfunction: An abridged Cochrane review
Source: BJU Int. 2026 Mar 30;137(6):949–57. doi: 10.1111/bju.70236 (PMC13168926; doi:10.1111/bju.70236)
Supplement: Supplementary file 2 — Table S1. Characteristics of included studies. [file BJU-137-949-s003.docx]

**Table S1. Characteristics of included studies**

**Ergun O, Kim K, Kim MH, Hwang EC, Blair Y, Gudeloglu A, Parekattil S, Dahm P**
<https://doi.org/10.1002/14651858.CD013166.pub3>

*The material in this section has been supplied by the author(s) for publication under a Licence for Publication and the author(s) are solely responsible for the material. Cochrane has reviewed this material, but Cochrane has not copyedited, formatted or proofread. Cochrane accordingly gives no representations or warranties of any kind in relation to, and accepts no liability for any reliance on or use of, such material.*

**Characteristics of included studies**

**Table of contents**

- [Studies ordered by Study ID](https://www.cochranelibrary.com/cdsr/doi/10.1002/14651858.CD013166.pub3/supplementarymaterials/CD013166-SUP-02-characteristicsOfIncludedStudies.html#studies)
  - [Chung 2022](https://www.cochranelibrary.com/cdsr/doi/10.1002/14651858.CD013166.pub3/supplementarymaterials/CD013166-SUP-02-characteristicsOfIncludedStudies.html#id444737038151864734)
  - [Fojecki 2017](https://www.cochranelibrary.com/cdsr/doi/10.1002/14651858.CD013166.pub3/supplementarymaterials/CD013166-SUP-02-characteristicsOfIncludedStudies.html#id444737038151864719)
  - [Harish 2017](https://www.cochranelibrary.com/cdsr/doi/10.1002/14651858.CD013166.pub3/supplementarymaterials/CD013166-SUP-02-characteristicsOfIncludedStudies.html#id444737038151864730)
  - [Kalyvianakis 2017](https://www.cochranelibrary.com/cdsr/doi/10.1002/14651858.CD013166.pub3/supplementarymaterials/CD013166-SUP-02-characteristicsOfIncludedStudies.html#id444737038151864731)
  - [Kalyvianakis 2022](https://www.cochranelibrary.com/cdsr/doi/10.1002/14651858.CD013166.pub3/supplementarymaterials/CD013166-SUP-02-characteristicsOfIncludedStudies.html#id444737038151864721)
  - [Kennady 2023](https://www.cochranelibrary.com/cdsr/doi/10.1002/14651858.CD013166.pub3/supplementarymaterials/CD013166-SUP-02-characteristicsOfIncludedStudies.html#id446430335802302509)
  - [Kim 2020](https://www.cochranelibrary.com/cdsr/doi/10.1002/14651858.CD013166.pub3/supplementarymaterials/CD013166-SUP-02-characteristicsOfIncludedStudies.html#id444737038151864723)
  - [Kitrey 2016](https://www.cochranelibrary.com/cdsr/doi/10.1002/14651858.CD013166.pub3/supplementarymaterials/CD013166-SUP-02-characteristicsOfIncludedStudies.html#id444737038151864733)
  - [Olsen 2015](https://www.cochranelibrary.com/cdsr/doi/10.1002/14651858.CD013166.pub3/supplementarymaterials/CD013166-SUP-02-characteristicsOfIncludedStudies.html#id444737038151864716)
  - [Ong 2022](https://www.cochranelibrary.com/cdsr/doi/10.1002/14651858.CD013166.pub3/supplementarymaterials/CD013166-SUP-02-characteristicsOfIncludedStudies.html#id444737038151864718)
  - [Ortac 2021](https://www.cochranelibrary.com/cdsr/doi/10.1002/14651858.CD013166.pub3/supplementarymaterials/CD013166-SUP-02-characteristicsOfIncludedStudies.html#id444737038151864727)
  - [Shendy 2021](https://www.cochranelibrary.com/cdsr/doi/10.1002/14651858.CD013166.pub3/supplementarymaterials/CD013166-SUP-02-characteristicsOfIncludedStudies.html#id444737038151864720)
  - [Spivak 2018](https://www.cochranelibrary.com/cdsr/doi/10.1002/14651858.CD013166.pub3/supplementarymaterials/CD013166-SUP-02-characteristicsOfIncludedStudies.html#id444737038151864725)
  - [Sramkova 2020](https://www.cochranelibrary.com/cdsr/doi/10.1002/14651858.CD013166.pub3/supplementarymaterials/CD013166-SUP-02-characteristicsOfIncludedStudies.html#id444737038151864724)
  - [Srini 2015](https://www.cochranelibrary.com/cdsr/doi/10.1002/14651858.CD013166.pub3/supplementarymaterials/CD013166-SUP-02-characteristicsOfIncludedStudies.html#id444737038151864729)
  - [Vardi 2012](https://www.cochranelibrary.com/cdsr/doi/10.1002/14651858.CD013166.pub3/supplementarymaterials/CD013166-SUP-02-characteristicsOfIncludedStudies.html#id444737038151864717)
  - [Vinay 2017](https://www.cochranelibrary.com/cdsr/doi/10.1002/14651858.CD013166.pub3/supplementarymaterials/CD013166-SUP-02-characteristicsOfIncludedStudies.html#id444737038151864722)
  - [Vinay 2021](https://www.cochranelibrary.com/cdsr/doi/10.1002/14651858.CD013166.pub3/supplementarymaterials/CD013166-SUP-02-characteristicsOfIncludedStudies.html#id444737038151864732)
  - [Xin 2017](https://www.cochranelibrary.com/cdsr/doi/10.1002/14651858.CD013166.pub3/supplementarymaterials/CD013166-SUP-02-characteristicsOfIncludedStudies.html#id444737038151864735)
  - [Yang 2019](https://www.cochranelibrary.com/cdsr/doi/10.1002/14651858.CD013166.pub3/supplementarymaterials/CD013166-SUP-02-characteristicsOfIncludedStudies.html#id444737038151864728)
  - [Yee 2014](https://www.cochranelibrary.com/cdsr/doi/10.1002/14651858.CD013166.pub3/supplementarymaterials/CD013166-SUP-02-characteristicsOfIncludedStudies.html#id444737038151864726)
- [Footnotes](https://www.cochranelibrary.com/cdsr/doi/10.1002/14651858.CD013166.pub3/supplementarymaterials/CD013166-SUP-02-characteristicsOfIncludedStudies.html#footnotes)
- [References to studies](https://www.cochranelibrary.com/cdsr/doi/10.1002/14651858.CD013166.pub3/supplementarymaterials/CD013166-SUP-02-characteristicsOfIncludedStudies.html#references)

**Studies ordered by Study ID**

| **Chung 2022** | | |
| --- | --- | --- |
| ***Study characteristics*** | | |
| Methods | Study design: randomized double-blind placebo controlled trial  Study setting/country: single center/Australia  Study period: January 2018 to January 2019 | |
| Participants | Inclusion criteria: patient age ≥ 18 years, has a poor response to medical therapy, a minimum 6-month history of ED, IIEF-5 score ≥ 12, and is in a stable sexual relationship for more than 3 months  Exclusion criteria: patients who developed ED following prostate cancer treatment such as prostatectomy or radiation, or pre-existing anatomical or neurological conditions  Total number of participants randomized: 60  Group A (LiSWT)  Number of participants assigned: 30  Age (SD): 45 (median) (range 42-68)  IIEF (SD): 14.6 (3.8)  EHS (SD): 1.4 (0.6)  ED Duration (SD): 33 months (median) (range 8-59)  Sexual QoL (SD): NA  Group B (sham)  Number of participants assigned: 30  Age (SD): 48 (median) (range 42-63)  IIEF (SD): 14.8 (3.6)  EHS (SD): 1.3 (0.7)  ED Duration (SD): 35 months (median) (range 6-60)  Sexual QoL (SD): NA | |
| Interventions | Group A  Device: second generation Duolith SD1 ultra (Storz Medical AG, Tägerwilen, Switzerland)  Frequency or dose: number of shocks: 3000 shockwaves; energy: 0.25 mJ/mm2; frequency: 6 Hz; anesthesia: no local or systemic analgesia; schedule: 12 treatments over six weeks (2 sessions per week)  Group B  Device: sham treatment was performed using the same medical device and handpiece as in the active LI-ESWT with the only difference, namely a standoff device at the end of the handpiece that does not transmit any shock waves  Frequency or dose: same frequency | |
| Outcomes | Primary outcomes: IIEF-5 questionnaire, adverse events  Time point measured: baseline, 1, 3, and 6 months after last treatment  Secondary outcomes: EHS, EDITS  Time point measured: baseline, 1, 3, and 6 months after last treatment | |
| Funding sources | Storz Medical AG | |
| Declarations of interest | None | |
| Notes | Language of publication: English  Type of publication: full-text article  Date of communication with authors: 17 October 2023  Contact status: reply by the author; based on author feedback, we re-evaluated performance bias and detection bias. Due to sufficient information lowering our concerns about these biases, we changed them from unclear to low. | |
| ***Risk of bias*** | | |
| **Bias** | **Authors' judgement** | **Support for judgement** |
| Random sequence generation (selection bias) | Low risk | Quote: "Patients were randomized using computer generated random list" |
| Allocation concealment (selection bias) | Unclear risk | Judgment: allocation concealment was not described in detail |
| Blinding (performance bias and detection bias) Blinding of participants and personnel (performence bias) | Low risk | Quote: "both the clinicians and patients (e-mail)" Judgment: no information who were blinded |
| Blinding (performance bias and detection bias) Blinding of outcome assessor (detection bias) | Low risk | Quote: "An independent third party is involved in collecting the data. Hence, the outcome assessor also blinded" |
| Incomplete outcome data (attrition bias) Erectile function | Low risk | Judgment: all randomized participants were included in the analysis |
| Incomplete outcome data (attrition bias) Discontinuation from treatment | Low risk | Judgment: all randomized participants were included in the analysis |
| Incomplete outcome data (attrition bias) Treatment-related adverse events | Low risk | Judgment: all randomized participants were included in the analysis |
| Incomplete outcome data (attrition bias) Quality of sexual life | Unclear risk | Judgment: no information |
| Incomplete outcome data (attrition bias) Patient/partner satisfaction | Unclear risk | Judgment: no information |
| Incomplete outcome data (attrition bias) Penile rigidity | Low risk | Judgment: all randomized participants were included in the analysis |
| Selective reporting (reporting bias) | Unclear risk | Judgment: the protocol was not found |
| Other bias | Low risk | Judgment: not found |

| **Fojecki 2017** | | |
| --- | --- | --- |
| ***Study characteristics*** | | |
| Methods | Study design: randomized, double blinded sham controlled study  Study setting/country: likely multicenter/Denmark  Study period: February 2014 to August 2014 | |
| Participants | Inclusion criteria: men aged over 40 years old, IIEF < 25, ED > 6 months, in stable relationship > 3 months  Exclusion criteria: men with surgery/radiotherapy of pelvic region, anticoagulants except acetylsalicylic acid 75mg, antiandrogens, anatomic penile deformation or penile prosthesis, total testosterone <8nmol/dl, serious heart or lung disease, psychiatric or neurologic disorder, pregnant partner, IIEF-EF ≥ 25  Total number of participants randomized: 126  Group A (LiSWT)  Number of participants assigned: 63  Age (SD): 65.4 (3.8)  IIEF (SD): 10.9 (mean) (95% CI: 9.1-12.7)  EHS (SD): NA  ED Duration (SD): NA  Sexual QoL (SD): 43.2 (likely mean) (95% CI: 36.2-50.2)  Group B (sham)  Number of participants assigned: 63  Age (SD): 63.3 (9.5)  IIEF (SD): 11.5 (mean) (95% CI: 9.8-13.2)  EHS (SD): NA  ED Duration (SD): NA  Sexual QoL (SD): 41.7 (likely mean) (95% CI: 36.2-47.3) | |
| Interventions | Group A  Device: piezoelectric linear therapy source (FBL10, Richard-Wolf GmbH, Knitlingen, Germany)  Frequency or dose: number of shocks: 600 shockwaves; energy: 0.09 mJ/mm2; frequency: 5 Hz; anesthesia: not reported; schedule: two rounds of five weekly sessions  Group B  Device: sham treatment; placebo gel pad will prevent shockwave emission  Frequency or dose: same frequency | |
| Outcomes | Primary outcomes: IIEF-EF score, adverse events  Time point measured: baseline, 4 weeks after last treatment  Secondary outcomes: EHS, Sexual Quality of Life–Men, EDITS  Time point measured: baseline, and nine weeks (four weeks after five weekly treatment sessions) | |
| Funding sources | None | |
| Declarations of interest | None | |
| Notes | Language of publication: English  Type of publication: full-text article  Date of communication with authors: 16 October 2023  Contact status: reply by the author; data provided | |
| ***Risk of bias*** | | |
| **Bias** | **Authors' judgement** | **Support for judgement** |
| Random sequence generation (selection bias) | Low risk | Quote: "A random list of numbers was generated (http://www. randomization.com)" |
| Allocation concealment (selection bias) | Unclear risk | Judgment: allocation concealment was not described in detail |
| Blinding (performance bias and detection bias) Blinding of participants and personnel (performence bias) | Low risk | Quote: "The subjects and the physician were blinded throughout the trial" |
| Blinding (performance bias and detection bias) Blinding of outcome assessor (detection bias) | Unclear risk | Judgment: no information whether outcome assessor was blinded |
| Incomplete outcome data (attrition bias) Erectile function | Low risk | Judgment: 58/63 (92.1%) randomized participants in experimental group and 60/63 (95.2%) in control group were included in the analysis |
| Incomplete outcome data (attrition bias) Discontinuation from treatment | Low risk | Judgment: nearly all randomized participants were included in the analysis |
| Incomplete outcome data (attrition bias) Treatment-related adverse events | Low risk | Judgment: 58/63 (92.1%) randomized participants in experimental group and 60/63 (95.2%) in control group were included in the analysis |
| Incomplete outcome data (attrition bias) Quality of sexual life | Low risk | Judgment: 58/63 (92.1%) randomized participants in experimental group and 60/63 (95.2%) in control group were included in the analysis |
| Incomplete outcome data (attrition bias) Patient/partner satisfaction | Unclear risk | Judgment: no information |
| Incomplete outcome data (attrition bias) Penile rigidity | Low risk | Judgment: 58/63 (92.1%) randomized participants in experimental group and 60/63 (95.2%) in control group were included in the analysis |
| Selective reporting (reporting bias) | Unclear risk | Judgment: study outcomes were well predefined and described, but not planned study outcomes (i.e. SQoL-M) were presented in the published full text article |
| Other bias | Low risk | Judgment: not found |

| **Harish 2017** | | |
| --- | --- | --- |
| ***Study characteristics*** | | |
| Methods | Study design: randomized, double-blind, sham controlled trial  Study setting/ country: single center/ India  Study period: NA | |
| Participants | Inclusion criteria: NA  Exclusion criteria: NA  Total number of participants randomized: 60  Group A (LiSWT)  Number of participants assigned: 30  Age (SD): NA  IIEF (SD): NA  EHS (SD): NA  ED Duration (SD): NA  Sexual QoL (SD): NA  Group B (sham)  Number of participants assigned: 30  Age (SD):NA  IIEF (SD): NA  EHS (SD): NA  ED Duration (SD): NA  Sexual QoL (SD): NA | |
| Interventions | Group A  Device: NA  Frequency or dose: NA  Group B  Device: NA  Frequency or dose: NA | |
| Outcomes | Primary outcomes: IIEF-EF questionnaire  Time point measured: baseline, 1 and 6 months  Primary outcomes: EHS scores  Time point measured: baseline, 1 and 6 months | |
| Funding sources | NA | |
| Declarations of interest | NA | |
| Notes | Language of publication: English  Type of publication: full-text article  Date of communication with authors: NA | |
| ***Risk of bias*** | | |
| **Bias** | **Authors' judgement** | **Support for judgement** |
| Random sequence generation (selection bias) | Unclear risk | Quote: "In this double-blind, placebo-controlled study" |
| Allocation concealment (selection bias) | Unclear risk | Judgment: allocation concealment was not described in detail |
| Blinding (performance bias and detection bias) Blinding of participants and personnel (performence bias) | Unclear risk | Judgment: no information who were blinded |
| Blinding (performance bias and detection bias) Blinding of outcome assessor (detection bias) | Unclear risk | Judgment: no information whether outcome assessor was blinded |
| Incomplete outcome data (attrition bias) Erectile function | Low risk | Judgment: all randomized participants were included in the analysis |
| Incomplete outcome data (attrition bias) Discontinuation from treatment | Low risk | Judgment: all randomized participants were included in the analysis |
| Incomplete outcome data (attrition bias) Treatment-related adverse events | Low risk | Judgment: all randomized participants were included in the analysis |
| Incomplete outcome data (attrition bias) Quality of sexual life | Unclear risk | Judgment: no information |
| Incomplete outcome data (attrition bias) Patient/partner satisfaction | Unclear risk | Judgment: no information |
| Incomplete outcome data (attrition bias) Penile rigidity | Low risk | Judgment: all randomized participants were included in the analysis |
| Selective reporting (reporting bias) | Unclear risk | Judgment: the protocol was not found |
| Other bias | Low risk | Judgment: not found |

| **Kalyvianakis 2017** | | |
| --- | --- | --- |
| ***Study characteristics*** | | |
| Methods | Study design: randomized, double-blind, sham controlled trial  Study setting/country: single center/Greece  Study period: NA | |
| Participants | Inclusion criteria: men who were at least 18 years old, had ED for at least 6 months, were at least partial responders to PDE5-I, had been stable heterosexual relationship with the same partner for more than 3 months, and had to be at least 6 to 21 in IIEF-EF  Exclusion criteria: men who had no ED or mild ED, radical prostatectomy, psychogenic ED, penile anatomic abnormalities, neurogenic ED, hormonal abnormalities  Total number of participants randomized: 46  Group A (LiSWT)  Number of participants assigned: 30  Age (SD): 53 (median) (range 31-72)  IIEF (SD): 13.8 (3.6)  EHS (SD): NA  ED Duration (SD): 5.5 (median) (range 1-20)  Sexual QoL (SD): NA  Group B (control)  Number of participants assigned: 16  Age (SD): 55.1 (median) (range 38-72)  IIEF (SD): 14.6 (3.4)  EHS (SD): NA  ED Duration (SD): 5.5 (median) (range 1-15)  Sexual QoL (SD): NA | |
| Interventions | Group A  Device: Omnispec ED 1000 electrohydraulic device (Medispec, Yehud, Israel)  Frequency or dose: number of shocks: 1500 shockwaves; energy: 0.09mJ/mm2; frequency: 160 pulses/min; anesthesia: none; schedule: 12 treatments over nine weeks  Group B  Device: sham treatment: the sham treatment was conducted using a distinctively designed shockwave applicator. The sham shockwave applicator contained an element that blocked delivery of shockwaves  Frequency or dose: NA | |
| Outcomes | Primary outcomes: IIEF-EF score, adverse events  Time point measured: baseline, 1, 3, 6, 9, and 12 months after last treatment  Secondary outcomes: PSV and RI  Time point measured: baseline, 3 months after last treatment | |
| Funding sources | Medispec | |
| Declarations of interest | Author received speaker honoraria from Medispec, Dornier Med Tech, and Menarini; and has research grants from Medispec and Dornier Med Tech. | |
| Notes | Language of publication: English  Type of publication: full-text article  Date of communication with authors: 14 October 2023  Contact status: no reply by the author | |
| ***Risk of bias*** | | |
| **Bias** | **Authors' judgement** | **Support for judgement** |
| Random sequence generation (selection bias) | Low risk | Quote: "All patients were blindly randomized using specific computer software into one of two active treatment groups or into a sham control group in a 2:1 ratio, respectively" |
| Allocation concealment (selection bias) | Unclear risk | Judgment: allocation concealment was not described in detail |
| Blinding (performance bias and detection bias) Blinding of participants and personnel (performence bias) | Unclear risk | Quote: "double-blinded fashion" Judgment: no information who were blinded |
| Blinding (performance bias and detection bias) Blinding of outcome assessor (detection bias) | Unclear risk | Judgment: no information whether outcome assessor was blinded |
| Incomplete outcome data (attrition bias) Erectile function | Low risk | Judgment: all randomized participants were included in the analysis |
| Incomplete outcome data (attrition bias) Discontinuation from treatment | Low risk | Judgment: all randomized participants were included in the analysis |
| Incomplete outcome data (attrition bias) Treatment-related adverse events | Low risk | Judgment: all randomized participants were included in the analysis |
| Incomplete outcome data (attrition bias) Quality of sexual life | Unclear risk | Judgment: no information |
| Incomplete outcome data (attrition bias) Patient/partner satisfaction | Unclear risk | Judgment: no information |
| Incomplete outcome data (attrition bias) Penile rigidity | Unclear risk | Judgment: no information |
| Selective reporting (reporting bias) | Unclear risk | Judgment: the protocol was not found |
| Other bias | Low risk | Judgment: not found |

| **Kalyvianakis 2022** | | |
| --- | --- | --- |
| ***Study characteristics*** | | |
| Methods | Study design: randomized, double-blind, sham controlled trial  Study setting/country: single center/Greece  Study period: June 2018 to July 2021 | |
| Participants | Inclusion criteria: 1) sexually active male patients 40–70 years old in a stable, heterosexual relationship for more than 3 months, 2) presence of vasculogenic ED (defined after medical history by experienced clinicians) for at least 6 months, 3) regular use of any PDE5 inhibitor with good or partial response to treatment (defined as at least 5/10 successful sexual intercourse attempts), 4) presence of moderate vasculogenic ED after a 1-month washout from PDE5 inhibitors, documented with questions 11-16 score in the IIEF-EF, 5) agreement to suspend any ED treatment for the duration of the study, 6) agreement to attempt sexual intercourse, without prior intake of alcohol or recreational drugs, at least 4 times every month for the duration of the study and document the outcome of each attempt using the SEP diaries.  Exclusion criteria: 1) any history of trauma, major surgery or radiation to the pelvis, 2) any history of priapism, penile fracture or major penile surgery, 3) presence of Peyronie’s disease or other anatomical disorders restricting sexual intercourse, 4) abnormal serum testosterone levels (<300 ng/dl or >1,197 ng/dl), 5) presence of any severe or unregulated medical or psychiatric disease precluding participation to the study, 6) allergy to the ultrasound gel, 7) partners of patients with self-reported sexual dysfunction or other medical conditions restricting sexual activity, 8) pregnant, breastfeeding or younger than 18 years old partners of patients.  Total number of participants randomized: 70  Group A (LiSWT)  Number of participants assigned: 35  Age: 54 (median) (47-63 IQR)  IIEF: 14 (median) (13-16 IQR)  EHS: NA  ED Duration: 68 (median) (35-124 IQR)  Sexual QoL: NA  Group B (Sham)  Number of participants assigned: 35  Age: 61 (median) (52-64 IQR)  IIEF: 15 (median) (13-16 IQR)  EHS: NA  ED Duration: 48 (median) (32-97 IQR)  Sexual QoL: NA | |
| Interventions | Group A  Device: ARIES 2TM and Smart Focus probe (Dornier MedTech GmbH, Wessling, Germany).  Frequency or dose: 5,000 impulses along the penis at an energy flux density of 0.096 mJ/mm2 and a frequency of 5 Hz (level 7 at the ARIES 2 generator). More specifically, 2,000 impulses were delivered to the corpora cavernosa, 2,000 to the crura cavernosa and 1,000 to the penile hila (based on a protocol developed by our research team) twice-weekly for 6 weeks.  Group B  Device: sham probe  Frequency or dose: Same frequency | |
| Outcomes | Primary outcomes: Proportion of patients in each group attaining MCID based on the IIEF- EF at 1 month and 3 months after completion of the treatment protocol, the mean change from baseline in the IIEF-EF between the 2 groups at the 1- and 3-month evaluations after completion of treatment protocol, any treatment related adverse events  Secondary outcomes: NA | |
| Funding sources | Dornier MedTech GmbH | |
| Declarations of interest | Author had financial interest and/or other relationship with Dornier MedTech GmbH | |
| Notes | Language of publication: English  Type of publication: full-text article  Date of communication with authors: NA | |
| ***Risk of bias*** | | |
| **Bias** | **Authors' judgement** | **Support for judgement** |
| Random sequence generation (selection bias) | Low risk | Quote: "Patients fulfilling the eligibility criteria underwent randomization, based on a computer-generated simple randomization sequence, in a 1:1 ratio to 12 sessions of LiST or sham therapy twice-weekly for 6 weeks. To preserve allocation concealment, the coordinating team performed the assignment of all patients to each group via a web-based registration system." |
| Allocation concealment (selection bias) | Low risk | Quote: "Patients fulfilling the eligibility criteria underwent randomization, based on a computer-generated simple randomization sequence, in a 1:1 ratio to 12 sessions of LiST or sham therapy twice-weekly for 6 weeks. To preserve allocation concealment, the coordinating team performed the assignment of all patients to each group via a web-based registration system." |
| Blinding (performance bias and detection bias) Blinding of participants and personnel (performence bias) | Low risk | Quote: "physicians, staff collecting data, and patients were blinded to group allocation throughout the course of the study." |
| Blinding (performance bias and detection bias) Blinding of outcome assessor (detection bias) | Low risk | Quote: "physicians, staff collecting data, and patients were blinded to group allocation throughout the course of the study." |
| Incomplete outcome data (attrition bias) Erectile function | Low risk | Judgment: nearly all randomized participants were included in the analysis |
| Incomplete outcome data (attrition bias) Discontinuation from treatment | Low risk | Judgment: nearly all randomized participants were included in the analysis |
| Incomplete outcome data (attrition bias) Treatment-related adverse events | Low risk | Judgment: nearly all randomized participants were included in the analysis |
| Incomplete outcome data (attrition bias) Quality of sexual life | Unclear risk | Judgment: no information about the number of participants in the analysis |
| Incomplete outcome data (attrition bias) Patient/partner satisfaction | Unclear risk | Judgment: no information about the number of participants in the analysis |
| Incomplete outcome data (attrition bias) Penile rigidity | Low risk | Judgment: nearly all randomized participants were included in the analysis |
| Selective reporting (reporting bias) | Low risk | Judgment: study outcomes were well predefined and described, and protocol was found |
| Other bias | Low risk | Judgment: not found |

| **Kennady 2023** | | |
| --- | --- | --- |
| ***Study characteristics*** | | |
| Methods | Study design: randomized, single-blinded, sham controlled, crossover trial  Study setting/country: single center/USA  Study period: NR | |
| Participants | Inclusion criteria: 1) SHIM scores (≥8 and ≤21), 2) hemoglobin A1c (≤7.5% within 3 months), 3) testosterone levels (>300 ng/dL).  Exclusion criteria: 1) A history of extensive pelvic surgery, 2) non–prostate-related cancer treatment within the previous 6 months, 3) any past prostate cancer treatment, 4) significant neurologic disease, 5) known penile malformation (eg, Peyronie’s disease).  Total number of participants randomized: 33  Group A (LiSWT)  Number of participants assigned: 17  Age: 67 (median) (47.2-69.6 IQR)  IIEF-5: 10.8 (3.6)  EHS: NA  ED Duration: NA  Sexual QoL: NA  Group B (Sham)  Number of participants assigned: 16  Age: 64.6 (median) (52.3-70.2 IQR)  IIEF-5: 12.1 (3.3)  EHS: NA  ED Duration: NA  Sexual QoL: NA | |
| Interventions | Group A  Device: Treatments were performed with a handheld Duolith device (Storz).  Frequency or dose: 3,000 impulses along the penis at an energy flux density of 0.1 mJ/mm2 and a frequency of 5 Hz. More specifically, 2,000 impulses were delivered to the corpora cavernosa and 1,000 to the proximal corpus at the level of the perineum twice-weekly for 3 weeks.  Group B  Device: sham probe  Frequency or dose: Same frequency | |
| Outcomes | Primary outcomes: change in IIEF-5  Secondary outcomes: change in EHS | |
| Funding sources | None | |
| Declarations of interest | None | |
| Notes | Language of publication: English  Type of publication: full-text article  Date of communication with authors: NA | |
| ***Risk of bias*** | | |
| **Bias** | **Authors' judgement** | **Support for judgement** |
| Random sequence generation (selection bias) | Low risk | Quote: "Randomization was performed by permutated block randomization; block sizes ranged from 2 to 8 via the blockrand package in R stratified by clinical group. Electronic randomization was generated by the study biostatistician." |
| Allocation concealment (selection bias) | Unclear risk | Judgment: allocation concealment was not described in detail |
| Blinding (performance bias and detection bias) Blinding of participants and personnel (performence bias) | High risk | Quote: "the clinician performing the treatment had access to the randomization module, with other study personnel blinded to the arm." Quote: "Of note, while participants were blinded, all authors, providers, and statisticians were unblinded to grouping." |
| Blinding (performance bias and detection bias) Blinding of outcome assessor (detection bias) | High risk | Quote: "Of note, while participants were blinded, all authors, providers, and statisticians were unblinded to grouping." |
| Incomplete outcome data (attrition bias) Erectile function | Low risk | Judgment: all randomized participants were included in the analysis |
| Incomplete outcome data (attrition bias) Discontinuation from treatment | Unclear risk | Judgment: no information |
| Incomplete outcome data (attrition bias) Treatment-related adverse events | Unclear risk | Judgment: no information |
| Incomplete outcome data (attrition bias) Quality of sexual life | Unclear risk | Judgment: no information |
| Incomplete outcome data (attrition bias) Patient/partner satisfaction | Unclear risk | Judgment: no information |
| Incomplete outcome data (attrition bias) Penile rigidity | Unclear risk | Judgment: no information |
| Selective reporting (reporting bias) | Unclear risk | Judgment: the protocol was not found |
| Other bias | Low risk | Judgment: no information |

| **Kim 2020** | | |
| --- | --- | --- |
| ***Study characteristics*** | | |
| Methods | Study design: sham-controlled, double-blind, randomized prospective study  Study setting/country: likely multicenter/South Korea  Study period: 2017 to 2018 | |
| Participants | Inclusion criteria: men aged ≥ 20 clinically diagnosed mild or moderate ED for at least 6 months and a stable sexual relationship for > 3 months  Exclusion criteria: men with severe ED, psychogenic ED, neurological pathology, a history of prior radical prostatectomy, rectal extirpation, radiation therapy to the pelvic area, no anatomical malformation, heart disease inhibiting sexual contact, and medication with anticoagulant  Total number of participants randomized: 96  Group A (LiSWT)  Number of participants assigned: 46  Age (SD): 65.4 (3.8)  IIEF (SD): 16.6 (3)  EHS (SD): 2.5 (0.9)  ED Duration (SD): NA  Sexual QoL (SD): NA  Group B (control)  Number of participants assigned: 49  Age (SD): 65.1 (7.9)  IIEF (SD): 16.7 (3.2)  EHS (SD): 2.5 (0.8)  ED Duration (SD): NA  Sexual QoL (SD): NA | |
| Interventions | Group A  Device: MT 2000H (Urontech Korea, Hwaseong, Korea)  Frequency or dose: number of shocks: 3000 shockwaves; energy: 12-20 mJ/mm2; frequency: 5 Hz; anesthesia: not reported; schedule: two sessions every week for 3 weeks, repeated after a 3-week resting period  Group B  Device: for the sham treatment, the same probe as that in Li-ESWT was used  Frequency or dose: the energy was set to 0 during each treatment, and a similar noise was delivered to the patients during the procedure | |
| Outcomes | Primary outcomes: IIEF-EF score, adverse events  Time point measured: baseline, 4 and 7 weeks after last treatment  Secondary outcomes: EHS, SEPQ-2, SEPQ-3  Time point measured: baseline, 4 and 7 weeks after last treatment | |
| Funding sources | Ministry of Health & Welfare, Korea (grant HI17C1944) | |
| Declarations of interest | None | |
| Notes | Language of publication: English  Type of publication: full-text article  Date of communication with authors: 16 October 2023  Contact status: no reply by the author | |
| ***Risk of bias*** | | |
| **Bias** | **Authors' judgement** | **Support for judgement** |
| Random sequence generation (selection bias) | Low risk | Quote: "After completing a baseline measurement visit, participants (1:1) were allocated to the sham group or Li-ESWT group using stratified block randomization method." |
| Allocation concealment (selection bias) | Unclear risk | Judgment: allocation concealment was not described in detail |
| Blinding (performance bias and detection bias) Blinding of participants and personnel (performence bias) | Unclear risk | Judgment: no information who were blinded |
| Blinding (performance bias and detection bias) Blinding of outcome assessor (detection bias) | Unclear risk | Judgment: no information whether outcome assessor was blinded |
| Incomplete outcome data (attrition bias) Erectile function | Low risk | Judgment: all randomized participants were included in the analysis |
| Incomplete outcome data (attrition bias) Discontinuation from treatment | Low risk | Judgment: all randomized participants were included in the analysis |
| Incomplete outcome data (attrition bias) Treatment-related adverse events | Low risk | Judgment: all randomized participants were included in the analysis |
| Incomplete outcome data (attrition bias) Quality of sexual life | Unclear risk | Judgment: no information |
| Incomplete outcome data (attrition bias) Patient/partner satisfaction | Unclear risk | Judgment: no information |
| Incomplete outcome data (attrition bias) Penile rigidity | Low risk | Judgment: all randomized participants were included in the analysis |
| Selective reporting (reporting bias) | Unclear risk | Judgment: the protocol was not found |
| Other bias | Low risk | Judgment: not found |

| **Kitrey 2016** | | |
| --- | --- | --- |
| ***Study characteristics*** | | |
| Methods | Study design: randomized, double-blind, sham controlled study  Study setting/country: single center/Israel  Study period: NA | |
| Participants | Inclusion criteria: men who had stopped using PDE5 due to lack of efficacy less than 12 months before screening and could not achieve erection hard enough for vaginal penetration after electing to receive the dose of PDE5-I (EHS 2 or less)  Exclusion criteria: men with any penile anatomical abnormality, an unstable medical condition, or neurological or hormonal abnormalities, or they were being treated for prostate cancer  Total number of participants randomized: 58  Group A (LiSWT)  Number of participants assigned: 40  Age: 60 (median) (range 28-78)  IIEF-EF: 7 (median) (range 6-12)  EHS: NA  ED Duration: 60 (median) (range 11-240)  Sexual QoL: NA  Group B (control)  Number of participants assigned: 18  Age: 64 (median) (range 29-81)  IIEF-EF: 8 (median) (range 6-12)  EHS: NA  ED Duration: 72 (median) (range 8-180)  Sexual QoL: NA | |
| Interventions | Group A  Device: Omnispec ED 1000 electrohydraulic device (Medispec, Yehud, Israel)  Frequency or dose: number of shocks: 1500 shockwaves; energy: 0.09mJ/mm2; frequency: 2 Hz; anesthesia: none; schedule: 12 treatments over nine weeks (2 sessions per week) with a 3 week no treatment interval  Group B  Device: sham probe  Frequency or dose: same frequency | |
| Outcomes | Primary outcomes: improvement on IIEF-EF according to MCID criteria (>7 points for severe ED and 5 points for moderate ED), adverse events  Time point measured: baseline, 4 weeks after last treatment  Secondary outcomes: EHS 3 or greater, QoL  Time point measured: baseline, 4 weeks after last treatment | |
| Funding sources | Medispec Ltd | |
| Declarations of interest | Author had financial interest and/or other relationship with Medispec | |
| Notes | Language of publication: English  Type of publication: full-text article  Date of communication with authors: 17 October 2023  Contact status: no reply by the author | |
| ***Risk of bias*** | | |
| **Bias** | **Authors' judgement** | **Support for judgement** |
| Random sequence generation (selection bias) | Unclear risk | Quote: "We performed a prospective, randomized, double-blind, sham controlled study of 86 men who underwent initial screening" |
| Allocation concealment (selection bias) | Unclear risk | Judgment: allocation concealment was not described in detail |
| Blinding (performance bias and detection bias) Blinding of participants and personnel (performence bias) | Low risk | Quote: "The operator and the patient were blinded to treatment type" |
| Blinding (performance bias and detection bias) Blinding of outcome assessor (detection bias) | Unclear risk | Judgment: no information whether outcome assessor was blinded |
| Incomplete outcome data (attrition bias) Erectile function | Low risk | Judgment: nearly all randomized participants were included in the analysis |
| Incomplete outcome data (attrition bias) Discontinuation from treatment | Low risk | Judgment: nearly all randomized participants were included in the analysis |
| Incomplete outcome data (attrition bias) Treatment-related adverse events | Low risk | Judgment: nearly all randomized participants were included in the analysis |
| Incomplete outcome data (attrition bias) Quality of sexual life | Unclear risk | Judgment: nearly all randomized participants were included in the analysis |
| Incomplete outcome data (attrition bias) Patient/partner satisfaction | Unclear risk | Judgment: no information about the number of participants in the analysis |
| Incomplete outcome data (attrition bias) Penile rigidity | Low risk | Judgment: nearly all randomized participants were included in the analysis |
| Selective reporting (reporting bias) | Low risk | Judgment: Study outcomes were well predefined and described |
| Other bias | Low risk | Judgment: not found |

| **Olsen 2015** | | |
| --- | --- | --- |
| ***Study characteristics*** | | |
| Methods | Study design: randomized, blinded, placebo-controlled study  Study setting/ country: likely multicenter/ Denmark  Study period: 2012 to 2013 | |
| Participants | Inclusion criteria: men with ED of organic origin who had responded to PDE5-I for more than 6 months, EHS less than 2 and IIEF-15) score less than 20, age 18 – 80 years and having been in a stable relationship for more than 3 months  Exclusion criteria: men with psychogenic ED, neurological pathology, history of prior radical prostatectomy, rectal extirpation, radiation to pelvic area and recovery from any cancer within the past 5 years, heart disease prohibiting sexual activity or taking medication with antiandrogens  Total number of participants randomized: 112  Group A (LiSWT)  Number of participants assigned: 51  Age: 59 (median)  IIEF: NA  EHS: NA  ED Duration: 57 (mean)  Sexual QoL: NA  Group B (control)  Number of participants assigned: 54  Age: 60 (median)  IIEF: NA  EHS: NA  ED Duration: 64 (mean)  Sexual QoL: NA | |
| Interventions | Group A  Device: Handheld duolith SD1 machine (Storz, Tagerwilen, Switzerland)  Frequency or dose: number of shocks: 600 shockwaves; Energy: 0.15 mJ/mm2; frequency: 5 Hz; anesthesia: not reported; schedule: five treatments over five weeks  Group B  Device: cap positioned over probe by Department Chair to prevent shocks from being delivered in the sham.  Frequency or dose: same frequency | |
| Outcomes | Primary outcomes: IIEF-EF score, adverse events  Time point measured: baseline, 5 weeks after last treatment  Secondary outcomes: EHS score  Time point measured: baseline, 5 weeks after last treatment | |
| Funding sources | Storz medical | |
| Declarations of interest | None | |
| Notes | Language of publication: English  Type of publication: full-text article  Date of communication with authors: 15 September 2023  Contact status: no reply by the author | |
| ***Risk of bias*** | | |
| **Bias** | **Authors' judgement** | **Support for judgement** |
| Random sequence generation (selection bias) | Low risk | Quote: "The randomization was done using a computer-generated list with random numbers" |
| Allocation concealment (selection bias) | Low risk | Quote: "a randomization list that was stored in a sealed envelope. Both the men and the physicians were blinded to the allocation" |
| Blinding (performance bias and detection bias) Blinding of participants and personnel (performence bias) | Low risk | Quote: "Both the men and the physicians were blinded to the allocation" |
| Blinding (performance bias and detection bias) Blinding of outcome assessor (detection bias) | Unclear risk | Judgment: no information whether outcome assessor was blinded |
| Incomplete outcome data (attrition bias) Erectile function | Low risk | Judgment: all randomized participants were included in the analysis |
| Incomplete outcome data (attrition bias) Discontinuation from treatment | Low risk | Judgment: all randomized participants were included in the analysis |
| Incomplete outcome data (attrition bias) Treatment-related adverse events | Low risk | Judgment: all randomized participants were included in the analysis |
| Incomplete outcome data (attrition bias) Quality of sexual life | Unclear risk | Judgment: no information about the number of participants in the analysis |
| Incomplete outcome data (attrition bias) Patient/partner satisfaction | Unclear risk | Judgment: no information about the number of participants in the analysis |
| Incomplete outcome data (attrition bias) Penile rigidity | Low risk | Judgment: all randomized participants were included in the analysis |
| Selective reporting (reporting bias) | Unclear risk | Judgment: the protocol was not found |
| Other bias | Low risk | Judgment: not found |

| **Ong 2022** | | |
| --- | --- | --- |
| ***Study characteristics*** | | |
| Methods | Study design: randomized, double-blind, sham controlled trial  Study setting/country: single center/Malaysia  Study period: August 2019 to December 2020 | |
| Participants | Inclusion criteria: patients aged 18 years and older, erectile dysfunction symptoms which are present more than 1 month, patients who voluntarily participated in the study by signing an informed consent, patients on ED medication who have withheld medications for a minimum of 2 weeks (drug washout period) prior to shock wave treatment  Exclusion criteria: uncorrected coagulation and bleeding disorders, active lesions or infections at the penis or pubic areas, erectile dysfunction of psychological origin, any psychiatric disorder, erectile dysfunction secondary to medications (use of corticosteroids, antipsychotic, antiandrogenic therapy, antidepressant, antiparkinsonians), spinal cord injury patients, bladder cancer, prostate cancer, active colon cancer patients, post radical prostatectomy or radical pelvic surgery patients, history of pelvic radiotherapy, penile implantation, patients with sickle cell anemia, endocrine diseases that occur with ED: acromegaly, gigantism, Addison's disease, hyperprolactinemia, androgenic deficiency, hypogonadism, patients with neurological diseases  Total number of participants randomized: 51  Group A (LiSWT)  Number of participants assigned: 27  Age (SD): 61 (10)  IIEF-5: 12 (median) (7 IQR)  EHS (SD): 2.07 (0.83)  ED Duration: 2 years (median) (3.5 IQR)  Sexual QoL: NA  Group B (control)  Number of participants assigned: 24  Age (SD): 55.5 (13)  IIEF-5: 10 (median) (8 IQR)  EHS (SD): 1.96 (0.69)  ED Duration: 2 years median (3 IQR)  Sexual QoL: NA | |
| Interventions | Group A  Device: Piezowave2, a shockwave device manufactured by Richard Wolf GmbH, Pforzheimer StraBe 32 Knittlingen Germany  Frequency or dose: 4 consecutive weeks of shock waves were applied with the following parameters: penetration depth of 10–15 mm, frequency of 8 Hz and intensity of 15–20; 2000 impulses distributed in the dorsum penis (both corpus cavernosum) and 2000 impulses were delivered to the perineal area (both crus penis). A total of 4000 impulses were delivered for each session.  Group B  Device: sham probe  Frequency or dose: same frequency | |
| Outcomes | Primary outcomes: IIEF-5, adverse events  Time point measured: baseline, 1, 3, and 6 months after last treatment  Secondary outcomes: EHS  Time point measured: baseline, 1, 3, and 6 months after last treatment | |
| Funding sources | Avro Medical Sd Bhd funding for open access article processing fee | |
| Declarations of interest | None | |
| Notes | Language of publication: English  Type of publication: full-text article  Date of communication with authors: 17 October 2023  Contact status: replied by the author; allocation process | |
| ***Risk of bias*** | | |
| **Bias** | **Authors' judgement** | **Support for judgement** |
| Random sequence generation (selection bias) | Low risk | Quote: "The randomization sequence was computer generated by the study coordinator" |
| Allocation concealment (selection bias) | Unclear risk | Quote: "Treatment allocation was carried out in the proper manner for allocation concealment and bias minimization" |
| Blinding (performance bias and detection bias) Blinding of participants and personnel (performence bias) | Low risk | Quote: "The subjects and clinicians who were responsible for the data collection were blinded to the treatment protocols of Arm 1 or Arm 2" |
| Blinding (performance bias and detection bias) Blinding of outcome assessor (detection bias) | Low risk | Quote: "The subjects and clinicians who were responsible for the data collection were blinded to the treatment protocols of Arm 1 or Arm 2" |
| Incomplete outcome data (attrition bias) Erectile function | Low risk | Judgment: all randomized participants were included in the analysis |
| Incomplete outcome data (attrition bias) Discontinuation from treatment | Low risk | Judgment: all randomized participants were included in the analysis |
| Incomplete outcome data (attrition bias) Treatment-related adverse events | Low risk | Judgment: all randomized participants were included in the analysis |
| Incomplete outcome data (attrition bias) Quality of sexual life | Unclear risk | Judgment: no information about the number of participants in the analysis |
| Incomplete outcome data (attrition bias) Patient/partner satisfaction | Unclear risk | Judgment: no information about the number of participants in the analysis |
| Incomplete outcome data (attrition bias) Penile rigidity | Low risk | Judgment: all randomized participants were included in the analysis |
| Selective reporting (reporting bias) | Unclear risk | Judgment: the protocol (NMRR-19-696-46324) was not found |
| Other bias | Low risk | Judgment: not found |

| **Ortac 2021** | | |
| --- | --- | --- |
| ***Study characteristics*** | | |
| Methods | Study design: randomized, placebo-controlled, single-blinded trial  Study setting/country: single center/Turkey  Study period: July 2016 to February 2018 | |
| Participants | Inclusion criteria: Age between 18 and 75 years and diagnosis of mild ED (IIEF-EF score= 17-25) being made at least six months prior to study inclusion and being confirmed by Penile Doppler ultrasonography at baseline examination  Exclusion criteria: Uncontrolled diabetes (HbA1C > 9%), testosterone deficiency (< 3 ng/dl), treatment with PDE-5 inhibitors and other erecting drugs during the first four weeks of the study and concomitant treatment with gonadotropin-releasing hormone and analogs, testosterone, antihypertensive, diuretic, antidepressant, sedative, neuroleptic, hypnotic and anti-epileptic drugs (within four months after study entry), as well as vitamin K antagonist (throughout the study) and corticosteroids (four weeks before treatment). Furthermore, patients with concomitant neurological, hematologic, cardiovascular, coagulatory disease, and cancer were excluded, as well as patients who had undergone shock wave treatment within six months before study entry and patients who had undergone pelvic surgery, radical prostatectomy, and transurethral prostate resection within four weeks before baseline examination  Total number of participants randomized: 66  Group A (LiSWT)  Number of participants assigned: 44  Age (SD): 42.32 (9.88)  IIEF-EF (SD): 20.32 (2.32)  EHS: NA  ED Duration: NA  Sexual QoL: NA  Group B (control)  Number of participants assigned: 22  Age (SD): 39.86 (11.64)  IIEF-EF (SD): 19.68 (1.55)  EHS: NA  ED Duration: NA  Sexual QoL: NA | |
| Interventions | Group A  Device: DUOLITH ® SD1 shock wave generator, equipped with a focused shock wave handpiece with a long stand-off device (Storz Medical AG, Tägerwilen, Switzerland).  Frequency or dose: number of shocks: 3000 shockwaves; energy: 0.2 mJ/mm2; frequency: 5Hz; anesthesia: no local or systemic analgesia; schedule: 42 treatments over four weeks  Group B  Device: placebo treatment was identical to ESWT, except that the stand-off device of the ESWT handpiece was filled with a shock wave absorbent material.  Frequency or dose: same frequency | |
| Outcomes | Primary outcomes: IIEF-EF, device-related side effects  Time point measured: baseline, 3 months, 12 months  Secondary outcomes: NA  Time point measured: baseline, 3 months, 12 months | |
| Funding sources | Storz medical | |
| Declarations of interest | None | |
| Notes | Language of publication: English  Type of publication: full-text article  Date of communication with authors: 17 October 2023  Contact status: no reply by the author | |
| ***Risk of bias*** | | |
| **Bias** | **Authors' judgement** | **Support for judgement** |
| Random sequence generation (selection bias) | Unclear risk | Quote: "patients were randomized in a 2:1 ratio to ESWT or placebo treatment" |
| Allocation concealment (selection bias) | Unclear risk | Judgment: allocation concealment was not described in detail |
| Blinding (performance bias and detection bias) Blinding of participants and personnel (performence bias) | High risk | Quote: "All patients were blinded to their treatment allocation", "single blinded", personnel likely not blinded |
| Blinding (performance bias and detection bias) Blinding of outcome assessor (detection bias) | High risk | Quote: "All patients were blinded to their treatment allocation", "single blinded" |
| Incomplete outcome data (attrition bias) Erectile function | High risk | Judgment: All patients were lost to follow-up at 12 months for placebo group versus 19 remaining patients in intervention group |
| Incomplete outcome data (attrition bias) Discontinuation from treatment | Unclear risk | Judgment: no information |
| Incomplete outcome data (attrition bias) Treatment-related adverse events | Low risk | Judgment: all randomized participants were included in the analysis |
| Incomplete outcome data (attrition bias) Quality of sexual life | Unclear risk | Judgment: no information about the number of participants in the analysis |
| Incomplete outcome data (attrition bias) Patient/partner satisfaction | Unclear risk | Judgment: no information about the number of participants in the analysis |
| Incomplete outcome data (attrition bias) Penile rigidity | Unclear risk | Judgment: no information about the number of participants in the analysis |
| Selective reporting (reporting bias) | Unclear risk | Judgment: the protocol (2014-MDD-CE-55) was not found |
| Other bias | Low risk | Judgment: not found |

| **Shendy 2021** | | |
| --- | --- | --- |
| ***Study characteristics*** | | |
| Methods | Study design: randomized double-blinded controlled trial  Study setting/country: multi-center /Egypt  Study period: July 2018 to January 2020 | |
| Participants | Inclusion criteria: Adult patients with body mass index <30 kg/m2, suffering from type-2 DM diabetic neuropathy as confirmed by nerve conduction study, and mild to moderate ED lasting for at least six months  Exclusion criteria: Patients with a history of pelvic surgery, unstable medical or psychiatric disorders, neurological diseases other than diabetic neuropathy, chronic hematological diseases, or penile anatomical abnormalities and nonresponders to PDE5i  Total number of participants randomized: 42  Group A (LiSWT)  Number of participants assigned: 21  Age (SD): 48.6 (5.5)  IIEF-EF (SD): 12.75 (3.21)  EHS: NA  ED Duration: NA  Sexual QoL: NA  Group B (control)  Number of participants assigned: 21  Age (SD): 47.5 (5.7)  IIEF-EF (SD): 12.75 (2.61)  EHS: NA  ED Duration: NA  Sexual QoL: NA | |
| Interventions | Group A  Device: Chattanooga Intellect Focus Shockwave Therapy SKU: CSW-400 + PFE (Kegel exercises three times daily for 6 weeks)  Frequency or dose: number of shocks: 3000 shockwaves; energy: 0.09 mJ/mm2; frequency: not reported; anesthesia: no local or systemic analgesia; schedule: 12 treatments over nine weeks (2 sessions per week) with a 3 week no treatment interval  Group B  Device: PFE (Kegel exercises three times daily for 6 weeks) and sham therapy by a shock wave. The sham treatment was conducted using a distinctively designed shock wave applicator, which contains an element that blocked the delivery of shock waves.  Frequency or dose: same frequency | |
| Outcomes | Primary outcomes: IIEF-5, adverse events  Time point measured: baseline, 3 months after last treatment  Secondary outcomes: NA  Time point measured: baseline, 3 months after last treatment | |
| Funding sources | NA | |
| Declarations of interest | NA | |
| Notes | Language of publication: English  Type of publication: full-text article  Date of communication with authors: 17 October 2023  Contact status: no reply by the author | |
| ***Risk of bias*** | | |
| **Bias** | **Authors' judgement** | **Support for judgement** |
| Random sequence generation (selection bias) | Low risk | Quote: "Using the permuted block randomisation method according to a computer-generated list prepared by an independent statistician not involved with subject recruitment, the patients were allocated into two equal groups" |
| Allocation concealment (selection bias) | Unclear risk | Judgment: allocation concealment was not described in detail |
| Blinding (performance bias and detection bias) Blinding of participants and personnel (performence bias) | Unclear risk | Judgment: no information who were blinded |
| Blinding (performance bias and detection bias) Blinding of outcome assessor (detection bias) | Unclear risk | Judgment: no information who were blinded |
| Incomplete outcome data (attrition bias) Erectile function | Low risk | Judgment: all randomized participants were included in the analysis |
| Incomplete outcome data (attrition bias) Discontinuation from treatment | Low risk | Judgment: all randomized participants were included in the analysis |
| Incomplete outcome data (attrition bias) Treatment-related adverse events | Low risk | Judgment: all randomized participants were included in the analysis |
| Incomplete outcome data (attrition bias) Quality of sexual life | Unclear risk | Judgment: no information about the number of participants in the analysis |
| Incomplete outcome data (attrition bias) Patient/partner satisfaction | Unclear risk | Judgment: no information about the number of participants in the analysis |
| Incomplete outcome data (attrition bias) Penile rigidity | Unclear risk | Judgment: no information about the number of participants in the analysis |
| Selective reporting (reporting bias) | Unclear risk | Judgment: the protocol was not found |
| Other bias | Low risk | Judgment: not found |

| **Spivak 2018** | | |
| --- | --- | --- |
| ***Study characteristics*** | | |
| Methods | Study design: randomized, double-blind, sham controlled trial  Study setting/country: single center/Russia  Study period: NA | |
| Participants | Inclusion criteria: NA  Exclusion criteria: NA  Total number of participants randomized: 50  Group A (LiSWT)  Number of participants assigned: 36  Age (SD): NA  IIEF (SD): NA, SHIM score: 17 (mean)  EHS (SD): NA  ED Duration (SD): NA  Sexual QoL (SD): NA  Group B (sham)  Number of participants assigned: 14  Age (SD): NA  IIEF (SD): NA, SHIM score: 16 (mean)  EHS (SD): NA  ED Duration (SD): NA  Sexual QoL (SD): NA | |
| Interventions | Group A  Device: Medispec Model ED1000  Frequency or dose: Six sessions were applied twice a week in three consecutive weeks and repeated again after 3 weeks using Medispec Model ED1000 - in total 12 sessions. The intensity of shockwaves was predefined by the manufacturer. Patients received 300 shocks to each of the 5 locations: 3 spots along the penile shaft and 2 spots on the penile crura.  Group B  Device: sham treatment: NA  Frequency or dose: NA | |
| Outcomes | Primary outcomes: SHIM score  Time point measured: baseline, 1 and 3 months after last treatment  Secondary outcomes: NA | |
| Funding sources | None | |
| Declarations of interest | NA | |
| Notes | Language of publication: English  Type of publication: abstract  Date of communication with authors: NA | |
| ***Risk of bias*** | | |
| **Bias** | **Authors' judgement** | **Support for judgement** |
| Random sequence generation (selection bias) | Unclear risk | Quote: "prospective randomised double-blind sham-controlled study" |
| Allocation concealment (selection bias) | Unclear risk | Quote: "prospective randomised double-blind sham-controlled study" |
| Blinding (performance bias and detection bias) Blinding of participants and personnel (performence bias) | Low risk | Quote: "prospective randomised double-blind sham-controlled study", the blind team performed the procedure and collected all data from patients" |
| Blinding (performance bias and detection bias) Blinding of outcome assessor (detection bias) | Low risk | Quote: "the blind team performed the procedure and collected all data from patients" |
| Incomplete outcome data (attrition bias) Erectile function | Low risk | Judgment: all randomized participants were included in the analysis |
| Incomplete outcome data (attrition bias) Discontinuation from treatment | Low risk | Judgment: all randomized participants were included in the analysis |
| Incomplete outcome data (attrition bias) Treatment-related adverse events | Low risk | Judgment: all randomized participants were included in the analysis |
| Incomplete outcome data (attrition bias) Quality of sexual life | Unclear risk | Judgment: no information about the number of participants in the analysis |
| Incomplete outcome data (attrition bias) Patient/partner satisfaction | Unclear risk | Judgment: no information about the number of participants in the analysis |
| Incomplete outcome data (attrition bias) Penile rigidity | Unclear risk | Judgment: no information about the number of participants in the analysis |
| Selective reporting (reporting bias) | Unclear risk | Judgment: the protocol was not found |
| Other bias | Low risk | Judgment: not found |

| **Sramkova 2020** | | |
| --- | --- | --- |
| ***Study characteristics*** | | |
| Methods | Study design: single-blind, sham controlled randomized clinical trial  Study setting/country: single center/Czech Republic  Study period: September 2017 to March 2018 | |
| Participants | Inclusion criteria: men with mild to severe vasculogenic ED lasting for at least 6 months, had a stable partner, and regular sexual activity at least twice a week  Exclusion criteria: men with psychogenic and neurogenic ED (neurologic disease, pelvic surgery)  Total number of participants randomized: 60  Group A (LiSWT)  Number of participants assigned: 30  Age (SD): 53.9 (9.3)  IIEF (SD): 12.8 (3.9)  EHS (SD): 2.1 (0.5)  ED Duration (SD): 42 (range: 6 – 204)  Sexual QoL (SD): NA  Group B (control)  Number of participants assigned: 30  Age (SD): 54.7 (9.2)  IIEF (SD): 13.1 (3.6)  EHS (SD): 2.1 (0.5)  ED Duration (SD): 45 (range: 6 – 204)  Sexual QoL (SD): NA | |
| Interventions | Group A  Device: PiezoWave 2 unit (Richard Wolf GmbH and ELvation Medical GmbH) and the FBL10 × 5G2 linear focusing shockwave applicator  Frequency or dose:  Number of shocks: 6000 shockwaves/session; energy: 0.16 mJ/mm2; frequency: not reported; anesthesia: not reported; schedule: four sessions (2 sessions per week)  Group B  Device: sham treatment: in the placebo group, a special applicator probe was used with a gel head that blocked shockwaves  Frequency or dose: NA | |
| Outcomes | Primary outcomes: proportion of patients with an optimal treatment response (IIEF-5 ≥22), adverse events  Time point measured: baseline, 4 and 12 weeks after last treatment  Secondary outcomes: EHS, GAQ, SEP-2, SEP-3, patient’s and their partner’s subjective satisfaction  Time point measured: baseline, 4 and 12 weeks after last treatment | |
| Funding sources | NA | |
| Declarations of interest | None | |
| Notes | Language of publication: English  Type of publication: full-text article  Date of communication with authors: 14 October 2023  Contact status: no reply by the author | |
| ***Risk of bias*** | | |
| **Bias** | **Authors' judgement** | **Support for judgement** |
| Random sequence generation (selection bias) | Low risk | Quote: "Patients were randomized using the software from Sealed Envelope Ltd (www.sealedenvelope.com/simple-ran- domiser/v1/lists) into 2 age-matched groups (Group A: treatment; Group B: placebo) with 30 patients in each group" |
| Allocation concealment (selection bias) | Unclear risk | Judgment: allocation concealment was not described in detail |
| Blinding (performance bias and detection bias) Blinding of participants and personnel (performence bias) | High risk | Quote: "single-blinded", "patients could not know whether their treatment was a placebo" |
| Blinding (performance bias and detection bias) Blinding of outcome assessor (detection bias) | High risk | Quote: "single-blinded", "patients could not know whether their treatment was a placebo" |
| Incomplete outcome data (attrition bias) Erectile function | Low risk | Judgment: all randomized participants were included in the analysis |
| Incomplete outcome data (attrition bias) Discontinuation from treatment | Low risk | Judgment: all randomized participants were included in the analysis |
| Incomplete outcome data (attrition bias) Treatment-related adverse events | Low risk | Judgment: all randomized participants were included in the analysis |
| Incomplete outcome data (attrition bias) Quality of sexual life | Unclear risk | Judgment: no information |
| Incomplete outcome data (attrition bias) Patient/partner satisfaction | Low risk | Judgment: all randomized participants were included in the analysis |
| Incomplete outcome data (attrition bias) Penile rigidity | Low risk | Judgment: all randomized participants were included in the analysis |
| Selective reporting (reporting bias) | Unclear risk | Judgment: the protocol was not found |
| Other bias | Low risk | Judgment: not found |

| **Srini 2015** | | |
| --- | --- | --- |
| ***Study characteristics*** | | |
| Methods | Study design: randomized, double-blind, placebo-controlled trial  Study setting/country: single center/India  Study period: NR, screening period: September 2009 to September 2011 | |
| Participants | Inclusion criteria: men with ED for at least 6 months, IIEF-EF < 18 after a 4 week PDE5i washout, penile doppler to confirm organic ED, responders to PDE5i, stable heterosexual relationship of > 3 months, penile hemodynamics evaluated with real time ultrasonographic color doppler PSV < 30 cm despite PDE5i and visual sexual stimulation  Exclusion criteria: men who had a history of radical prostatectomy, pelvic radiotherapy, hormonal therapy, receiving treatment for psychiatric condition (penile doppler to exclude psychogenic ED), chronic hematological conditions, cardiovascular conditions that prevent sexual activity, history of heart attack, stroke or life-threatening arrhythmia within the previous 6 months, cancer within the past 5 years, use of any treatment for ED within 7 days of screening, anatomical, neurological or hormonal abnormalities  Total number of participants randomized: 135  Group A (LiSWT)  Number of participants assigned: 95  Age: NA  IIEF-EF: 9.5 (mean)  EHS: NA  ED Duration: NA, at least 6 months  Sexual QoL: NA  Group B (sham)  Number of participants assigned: 40  Age: NA  IIEF- EF: 9.2 (mean)  EHS: NA  ED Duration: NA, at least 6 months  Sexual QoL: NA | |
| Interventions | Group A  Device: Omnispec ED 1000 electrohydraulic device (Medispec, Yehud, Israel)  Frequency or dose: number of shocks: 300 shockwaves; energy: 0.15 mJ/mm2; frequency: 2 Hz; anesthesia: none; schedule: 12 treatments over nine weeks (2 sessions per week) with a 3 week no treatment interval  Group B  Device: For placebo, a metal plate was used to block the transmission.  Frequency or dose: same frequency | |
| Outcomes | Primary outcomes: 5-point or greater improvement in the IIEF-EF domain, adverse events  Time point measured: baseline, 6 weeks after randomization (1 week after treatment)  Secondary outcomes: EHS  Time point measured: baseline, 6 weeks after randomization (1 week after treatment) | |
| Funding sources | NA | |
| Declarations of interest | NA | |
| Notes | Language of publication: English  Type of publication: full-text article  Date of communication with authors: NA | |
| ***Risk of bias*** | | |
| **Bias** | **Authors' judgement** | **Support for judgement** |
| Random sequence generation (selection bias) | Unclear risk | Quote: "randomized, double-blind, placebo-controlled study" |
| Allocation concealment (selection bias) | Unclear risk | Quote: "randomized, double-blind, placebo-controlled study" |
| Blinding (performance bias and detection bias) Blinding of participants and personnel (performence bias) | Low risk | Quote: "both the operator and the subject were blinded to treatment randomization" |
| Blinding (performance bias and detection bias) Blinding of outcome assessor (detection bias) | Unclear risk | Judgment: no information whether outcome assessor was blinded |
| Incomplete outcome data (attrition bias) Erectile function | High risk | Judgment: 60/95 (63.2%) randomized participants in experimental group and 17/40 (42.5%) in control group were included in the analysis |
| Incomplete outcome data (attrition bias) Discontinuation from treatment | Low risk | Judgment: all randomized participants were included in the analysis |
| Incomplete outcome data (attrition bias) Treatment-related adverse events | High risk | Judgment: 60/95 (63.2%) randomized participants in experimental group and 17/40 (42.5%) in control group were included in the analysis |
| Incomplete outcome data (attrition bias) Quality of sexual life | Unclear risk | Judgment: no information about the number of participants in the analysis |
| Incomplete outcome data (attrition bias) Patient/partner satisfaction | Unclear risk | Judgment: no information about the number of participants in the analysis |
| Incomplete outcome data (attrition bias) Penile rigidity | High risk | Judgment: 60/95 (63.2%) randomized participants in experimental group and 17/40 (42.5%) in control group were included in the analysis |
| Selective reporting (reporting bias) | Unclear risk | Judgment: the protocol was not found |
| Other bias | Low risk | Judgment: not found |

| **Vardi 2012** | | |
| --- | --- | --- |
| ***Study characteristics*** | | |
| Methods | Study design: randomized double-blind placebo-controlled trial  Study setting/country: likely single center/Israel  Study period: July 2009 to October 2010 | |
| Participants | Inclusion criteria: men with IIEF-EF of 19 or greater while on PDE5-I, stable heterosexual relationship for more than 3 months, who agree to discontinue PDE5-I during the study period  Exclusion criteria: men with a history of radical prostatectomy, pelvic radiotherapy or hormonal therapy, were receiving ongoing treatment for a psychiatric condition, or had any anatomical, neurological or hormonal abnormalities  Total number of participants randomized: 67  Group A (LiSWT)  Number of participants assigned: 46  Age (SD): 58 (median) (range 27-72)  IIEF (SD): 12.6 (0.75)  EHS (SD): NA  ED Duration (SD): 42 (median) (range 6-240)  Sexual QoL (SD): NA  Group B (control)  Number of participants assigned: 21  Age (SD): 57 (median) (range 35-77)  IIEF (SD): 11.5 (0.86)  EHS (SD): NA  ED Duration (SD): 60 (median) (range 6-240)  Sexual QoL (SD): NA | |
| Interventions | Group A  Device: Omnispec ED 1000 electrohydraulic device (Medispec, Yehud, Israel)  Frequency or dose: number of shocks: 300 shockwaves; energy: 0.09 mJ/mm2; frequency: 2 Hz; anesthesia: not reported; schedule: 12 treatments over nine weeks (2 sessions per week) with a 3 week no treatment interval  Group B  Device: for sham treatment, a metal plate used to block the transmission.  Frequency or dose: likely same frequency | |
| Outcomes | Primary outcomes: IIEF-EF questionnaire (treatment success was defined as a 5-point or greater improvement in the IIEF-EF from baseline), adverse events  Time point measured: baseline, 4 weeks after last treatment  Secondary outcomes: EHS, penile blood flow  Time point measured: baseline, 4 weeks after last treatment | |
| Funding sources | Medispec, Ltd | |
| Declarations of interest | An author declared financial interest and/or other relationship with Medispec, Ltd. | |
| Notes | Language of publication: English  Type of publication: full-text article  Date of communication with authors: 15 September 2023  Contact status: no reply by the author | |
| ***Risk of bias*** | | |
| **Bias** | **Authors' judgement** | **Support for judgement** |
| Random sequence generation (selection bias) | Low risk | Quote: "At V1 the men were assigned into 2 groups of those who received LI-ESWT (treated group) and those who were given sham therapy (sham group) in a 2:1 ratio using a computer-generated table of random numbers." |
| Allocation concealment (selection bias) | Unclear risk | Judgment: allocation concealment was not described in detail |
| Blinding (performance bias and detection bias) Blinding of participants and personnel (performence bias) | Low risk | Quote: "operator and subject were blind to the treatment type." |
| Blinding (performance bias and detection bias) Blinding of outcome assessor (detection bias) | Unclear risk | Judgment: no information whether outcome assessor was blinded |
| Incomplete outcome data (attrition bias) Erectile function | High risk | Judgment: 40/46 (87.0%) randomized participants in experimental group and 20/21 (95.2%) in control group were included in the analysis |
| Incomplete outcome data (attrition bias) Discontinuation from treatment | Low risk | Judgment: all randomized participants were included in the analysis |
| Incomplete outcome data (attrition bias) Treatment-related adverse events | High risk | Judgment: 40/46 (87.0%) randomized participants in experimental group and 20/21 (95.2%) in control group were included in the analysis |
| Incomplete outcome data (attrition bias) Quality of sexual life | Unclear risk | Judgment: no information |
| Incomplete outcome data (attrition bias) Patient/partner satisfaction | Unclear risk | Judgment: no information |
| Incomplete outcome data (attrition bias) Penile rigidity | High risk | Judgment: 40/46 (87.0%) randomized participants in experimental group and 20/21 (95.2%) in control group were included in the analysis |
| Selective reporting (reporting bias) | Unclear risk | Judgment: the protocol was not found |
| Other bias | Low risk | Judgment: not found |

| **Vinay 2017** | | |
| --- | --- | --- |
| ***Study characteristics*** | | |
| Methods | Study design: randomized, simple blind, placebo-controlled trial  Study setting/country: single center/Spain  Study period: NA | |
| Participants | Inclusion criteria: NA  Exclusion criteria: NA  Total number of participants randomized: 58  Group A (LiSWT)  Number of participants assigned: 30  Age (SD): NA  IIEF (SD): 10 (3.9)  EHS (SD): NA  ED Duration (SD): NA  Sexual QoL (SD): NA  Group B (sham)  Number of participants assigned: 28  Age (SD): NA  IIEF (SD): 10 (4.5)  EHS (SD): NA  ED Duration (SD): NA  Sexual QoL (SD): NA | |
| Interventions | Group A  Device: electrohydraulic  Frequency or dose: 1 session/week for 6 weeks; 1,500 pulses of 0.10 mJ/mm2 at 5 Hz  Group B  Device: sham probe  Frequency or dose: NA | |
| Outcomes | Primary outcomes: IIEF-5  Time point measured: baseline and 1 month  Secondary outcomes: SEP question 2 and 3  Time point measured: baseline and 1 month | |
| Funding sources | None | |
| Declarations of interest | NA | |
| Notes | Language of publication: English  Type of publication: abstract  Date of communication with authors: 15 April 2023  Contact status: replied by the author; one full text record and two conference abstracts were based on the same population | |
| ***Risk of bias*** | | |
| **Bias** | **Authors' judgement** | **Support for judgement** |
| Random sequence generation (selection bias) | Unclear risk | Quote: "Prospective, randomized, simple-blind, sham-controlled study." |
| Allocation concealment (selection bias) | Unclear risk | Quote: "Prospective, randomized, simple-blind, sham-controlled study." |
| Blinding (performance bias and detection bias) Blinding of participants and personnel (performence bias) | High risk | Quote: "Prospective, randomized, simple-blind, sham-controlled study" Judgment: no information who were blinded |
| Blinding (performance bias and detection bias) Blinding of outcome assessor (detection bias) | Unclear risk | Quote: "Prospective, randomized, simple-blind, sham-controlled study" Judgment: no information who were blinded |
| Incomplete outcome data (attrition bias) Erectile function | High risk | Judgment: 27/30 (90.0%) randomized participants in experimental group and 20/28 (71.4%) in control group were included in the analysis |
| Incomplete outcome data (attrition bias) Discontinuation from treatment | Low risk | Judgment: all randomized participants were included in the analysis |
| Incomplete outcome data (attrition bias) Treatment-related adverse events | Unclear risk | Judgment: no information |
| Incomplete outcome data (attrition bias) Quality of sexual life | Unclear risk | Judgment: no information |
| Incomplete outcome data (attrition bias) Patient/partner satisfaction | Unclear risk | Judgment: no information |
| Incomplete outcome data (attrition bias) Penile rigidity | Unclear risk | Judgment: no information |
| Selective reporting (reporting bias) | Unclear risk | Judgment: the protocol was not found |
| Other bias | Low risk | Judgment: not found |

| **Vinay 2021** | | |
| --- | --- | --- |
| ***Study characteristics*** | | |
| Methods | Study design: prospective, randomized, double-blind, sham controlled trial  Study setting/country: single center/Spain  Study period: NA | |
| Participants | Inclusion criteria: men with history of ED of more than 6 months not responding to PDE5-I drugs (inadequate erectile response after at least four attempts using the highest tolerated drug)  Exclusion criteria: men with any penile anatomical abnormality, an unstable medical condition, neurological/hormonal abnormalities, history of pelvic surgery/radiotherapy, current use of psychotropic drugs or a diagnosis of a specific ED etiology different to vascular ED  Total number of participants randomized: 80  Group A (LiSWT)  Number of participants assigned: 40  Age (SD): 60 (median) (IQR 54-66)  IIEF (SD): 12 (median) (IQR 8-17)  EHS (SD): NA  ED Duration (SD): 3 (median) (IQR 2-6)  Sexual QoL (SD): NA  Group B (control)  Number of participants assigned: 40  Age (SD): 60 (median) (IQR 53-65)  IIEF (SD): 13 (median) (IQR 8-17)  EHS (SD): NA  ED Duration (SD): 4.5 (median) (IQR 3-6)  Sexual QoL (SD): NA | |
| Interventions | Group A  Device: RENOVA® electromagnetic device (Direx Group, Wiesbaden, Germany)  Frequency or dose: number of shocks: 5000 shockwaves; energy: 0.09 mJ/mm2; frequency: 2 Hz; anesthesia: not reported; Schedule: 1 session per week for 4 weeks  Group B  Device: the device probe was replaced with one that had the same shape, weight and sound  Frequency or dose: the probe did not generate shockwaves | |
| Outcomes | Primary outcomes: IIEF-EF score, adverse events  Time point measured: baseline, 1, 3, and 6 months after last treatment  Secondary outcomes: percentage of patients with an EHS > 2, positive answers to SEP2, SEP3 and GAQ1 questions  Time point measured: baseline, 1, 3, and 6 months after last treatment | |
| Funding sources | None | |
| Declarations of interest | The researchers received materials for the LI- EWST device from the manufacturer (Direx Group, Wiesbaden, Germany). | |
| Notes | Language of publication: English  Type of publication: full-text article  Date of communication with authors: 14 October 2023  Contact status: no reply by the author | |
| ***Risk of bias*** | | |
| **Bias** | **Authors' judgement** | **Support for judgement** |
| Random sequence generation (selection bias) | Low risk | Quote: "Patients who met study inclusion criteria were assigned in a 1:1 ratio (using a randomization software) to the active LI-ESWT group and the sham group" |
| Allocation concealment (selection bias) | Unclear risk | Judgment: allocation concealment was not described in detail |
| Blinding (performance bias and detection bias) Blinding of participants and personnel (performence bias) | Low risk | Quote: "Both patient and probe operator were blind to the procedure" |
| Blinding (performance bias and detection bias) Blinding of outcome assessor (detection bias) | Unclear risk | Judgment: no information whether outcome assessor was blinded |
| Incomplete outcome data (attrition bias) Erectile function | Low risk | Judgment: all randomized participants in experimental group and 36/40 (90.0%) in control group were included in the analysis |
| Incomplete outcome data (attrition bias) Discontinuation from treatment | Low risk | Judgment: all randomized participants were included in the analysis |
| Incomplete outcome data (attrition bias) Treatment-related adverse events | Low risk | Judgment: all randomized participants in experimental group and 36/40 (90.0%) in control group were included in the analysis |
| Incomplete outcome data (attrition bias) Quality of sexual life | Unclear risk | Judgment: no information |
| Incomplete outcome data (attrition bias) Patient/partner satisfaction | Unclear risk | Judgment: no information |
| Incomplete outcome data (attrition bias) Penile rigidity | Low risk | Judgment: all randomized participants in experimental group and 36/40 (90.0%) in control group were included in the analysis |
| Selective reporting (reporting bias) | Unclear risk | Judgment: the protocol was not found |
| Other bias | Low risk | Judgment: not found |

| **Xin 2017** | | |
| --- | --- | --- |
| ***Study characteristics*** | | |
| Methods | Study design: randomized, double-blind, sham controlled trial  Study setting/country: multicenter/China  Study period: NA | |
| Participants | Inclusion criteria: men with mild to moderate vasculogenic ED  Exclusion criteria: NA  Total number of participants randomized: 70  Group A (LiSWT)  Number of participants assigned: 46  Age: NA  IIEF: NA  EHS: NA  ED Duration: NA  Sexual QoL: NA  Group B (control)  Number of participants assigned: 24  Age: NA  IIEF: NA  EHS: NA  ED Duration: NA  Sexual QoL: NA | |
| Interventions | Group A  Device: NA  Frequency or dose: number of shocks: 5000 shockwaves; energy: NA; frequency: NA; anesthesia: not reported; schedule: one treatment session per week for 4 weeks  Group B  Device: sham device details are not available  Frequency or dose: sham device details are not available | |
| Outcomes | Primary outcomes: 5-point or greater improvement in the IIEF-EF from baseline  TIme point measured: at baseline, 1 and, 3 months after treatment  Secondary outcomes: EHS  TIme point measured: at baseline, 1 and, 3 months after treatment | |
| Funding sources | NA | |
| Declarations of interest | NA | |
| Notes | Language of publication: English  Type of publication: abstract  Date of communication with authors: 20 April 2023  Contact status: replied by the author; this study was from the trial registry (https://trialsearch.who.int/Trial2.aspx?TrialID=ChiCTR-INR-17014019 | |
| ***Risk of bias*** | | |
| **Bias** | **Authors' judgement** | **Support for judgement** |
| Random sequence generation (selection bias) | Unclear risk | Quote: "A multicenter, double-blinded, randomized sham-controlled clinical trial" |
| Allocation concealment (selection bias) | Unclear risk | Quote: "A multicenter, double-blinded, randomized sham-controlled clinical trial" |
| Blinding (performance bias and detection bias) Blinding of participants and personnel (performence bias) | Unclear risk | Quote: "A multicenter, double-blinded, randomized sham-controlled clinical trial" Judgment: no information who were blinded |
| Blinding (performance bias and detection bias) Blinding of outcome assessor (detection bias) | Unclear risk | Quote: "A multicenter, double-blinded, randomized sham-controlled clinical trial" Judgment: no information who were blinded |
| Incomplete outcome data (attrition bias) Erectile function | Low risk | Judgment: all randomized participants were included in the analysis |
| Incomplete outcome data (attrition bias) Discontinuation from treatment | Unclear risk | Judgment: no information about the number of participants in the analysis |
| Incomplete outcome data (attrition bias) Treatment-related adverse events | Low risk | Judgment: all randomized participants were included in the analysis |
| Incomplete outcome data (attrition bias) Quality of sexual life | Unclear risk | Judgment: no information about the number of participants in the analysis |
| Incomplete outcome data (attrition bias) Patient/partner satisfaction | Unclear risk | Judgment: no information about the number of participants in the analysis |
| Incomplete outcome data (attrition bias) Penile rigidity | Unclear risk | Judgment: no information about the number of participants in the analysis |
| Selective reporting (reporting bias) | High risk | Judgment: study outcomes were well predefined and described in the protocol, but some study outcomes were omitted in the abstract |
| Other bias | Low risk | Judgment: not found |

| **Yang 2019** | | |
| --- | --- | --- |
| ***Study characteristics*** | | |
| Methods | Study design: multi-center, double-blinded, prospective, randomized, sham-controlled trial  Study setting/country: multi-center/China  Study period: NA | |
| Participants | Inclusion criteria: NA  Exclusion criteria: NA  Total number of participants randomized: 60  Group A (LiSWT)  Number of participants assigned: 40  Age (SD): NA  IIEF (SD): NA  EHS (SD): NA  ED Duration (SD): NA  Sexual QoL (SD): NA  Group B (control)  Number of participants assigned: 20  Age (SD): NA  IIEF (SD): NA  EHS (SD): NA  ED Duration (SD): NA  Sexual QoL (SD): NA | |
| Interventions | Group A  Device: NA  Frequency or dose: number of shocks, energy, frequency, anesthesia: not reported; schedule: one treatment session per week for 4 weeks  Group B  Device: NA  Frequency or dose: NA | |
| Outcomes | Primary outcomes: IIEF-6 questionnaire, adverse events  Time point measured: baseline, 3 months after last treatment  Secondary outcomes: EHS/IAD, PSV, EDV, RI  Time point measured: baseline, 3 months after last treatment | |
| Funding sources | NA | |
| Declarations of interest | NA | |
| Notes | Language of publication: English  Type of publication: abstract  Date of communication with authors: NA | |
| ***Risk of bias*** | | |
| **Bias** | **Authors' judgement** | **Support for judgement** |
| Random sequence generation (selection bias) | Unclear risk | Quote: "This was a multi-center, double-blinded, prospective, randomized, placebo-controlled trial." |
| Allocation concealment (selection bias) | Unclear risk | Quote: "This was a multi-center, double-blinded, prospective, randomized, placebo-controlled trial." |
| Blinding (performance bias and detection bias) Blinding of participants and personnel (performence bias) | Unclear risk | Quote: "This was a multi-center, double-blinded, prospective, randomized, placebo-controlled trial." |
| Blinding (performance bias and detection bias) Blinding of outcome assessor (detection bias) | Unclear risk | Judgment: no information whether outcome assessor was blinded |
| Incomplete outcome data (attrition bias) Erectile function | Low risk | Judgment: 33/40 (82.5%) randomized participants in experimental group and 16/20 (80%) in control group were included in the analysis |
| Incomplete outcome data (attrition bias) Discontinuation from treatment | Low risk | Judgment: all randomized participants were included in the analysis |
| Incomplete outcome data (attrition bias) Treatment-related adverse events | Low risk | Judgment: all randomized participants were included in the analysis |
| Incomplete outcome data (attrition bias) Quality of sexual life | Unclear risk | Judgment: no information |
| Incomplete outcome data (attrition bias) Patient/partner satisfaction | Unclear risk | Judgment: no information |
| Incomplete outcome data (attrition bias) Penile rigidity | Unclear risk | Judgment: no information |
| Selective reporting (reporting bias) | Unclear risk | Judgment: the protocol was not found |
| Other bias | Low risk | Judgment: not found |

| **Yee 2014** | | |
| --- | --- | --- |
| ***Study characteristics*** | | |
| Methods | Study design: randomized, double-blind, sham-controlled trial  Study setting/country: single center/China  Study period: October 2011 to October 2012 | |
| Participants | Inclusion criteria: men aged ≥ 18 years old with more than a 6-month history of ED and heterosexual relationship, who scored ≤ 21 in the SHIM  Exclusion criteria: men with ED due to hypogonadism, androgen deprivation therapy, neurological disease, penile structural abnormality, radical prostatectomy or other pelvic surgery, pelvic irradiation, penile implant  Total number of participants randomized: 70  Group A (LiSWT)  Number of participants assigned: 36  Age (SD): 58.9 (7.6)  IIEF-EF (SD): 10.2 (3.8)  EHS (SD): 1.5 (0.6)  ED Duration (SD): 6.5 (2.8)  Sexual QoL: NA  Group B (control)  Number of participants assigned: 34  Age (SD): 63.3 (6.4)  IIEF-EF (SD): 10.2 (3.8)  EHS (SD): 1.4 (0.6)  ED Duration (SD): 7.4 (4.3)  Sexual QoL: NA | |
| Interventions | Group A  Device: Omnispec ED1000, Medispec, Germantown MD, USA  Frequency or dose: number of shocks: 1500 shockwaves; energy: 0.09 mJ/mm2; frequency: 2 Hz; anesthesia: no local or systemic analgesia; schedule: 12 treatments over nine weeks (2 sessions per week) with a 3 week no treatment interval  Group B  Device: Sham treatment: Energy setting was zero  Frequency or dose: same frequency | |
| Outcomes | Primary outcomes: IIEF-EF, adverse events  Time point measured: baseline, 4 weeks after last treatment  Secondary outcomes: EHS  Time point measured: baseline, 4 weeks after last treatment | |
| Funding sources | NA | |
| Declarations of interest | None | |
| Notes | Language of publication: English  Type of publication: full-text article  Date of communication with authors: 17 October 2023  Contact status: replied by the author; the details of allocation concealment and blinding | |
| ***Risk of bias*** | | |
| **Bias** | **Authors' judgement** | **Support for judgement** |
| Random sequence generation (selection bias) | Low risk | Quote: "They were assigned into either the treatment group (with Li-ESWT) or the sham group (sham therapy) in a 1:1 ratio using a computer-generated table of random numbers. The randomization process was in a block-size of two and four, without stratification." |
| Allocation concealment (selection bias) | Unclear risk | Judgment: allocation concealment was not described in detail |
| Blinding (performance bias and detection bias) Blinding of participants and personnel (performence bias) | Unclear risk | Quote: "All investigators and research assistants involved in the assessment of the participants were blind to group assignment. For patients with a history of PDE5I use, they underwent a 2-week washout period before the randomization process." Judgment: no information participants were blinded |
| Blinding (performance bias and detection bias) Blinding of outcome assessor (detection bias) | Low risk | Quote: "All investigators and research assistants involved in the assessment of the participants were blind to group assignment. For patients with a history of PDE5I use, they underwent a 2-week washout period before the randomization process." Judgment: no information participants were blinded |
| Incomplete outcome data (attrition bias) Erectile function | Unclear risk | Judgment: 30/36 (83.3%) randomized participants in experimental group and 28/34 (82.4%) in control group were included in the analysis |
| Incomplete outcome data (attrition bias) Discontinuation from treatment | Low risk | Judgment: all randomized participants were included in the analysis |
| Incomplete outcome data (attrition bias) Treatment-related adverse events | Unclear risk | Judgment: 30/36 (83.3%) randomized participants in experimental group and 28/34 (82.4%) in control group were included in the analysis |
| Incomplete outcome data (attrition bias) Quality of sexual life | Unclear risk | Judgment: no information |
| Incomplete outcome data (attrition bias) Patient/partner satisfaction | Unclear risk | Judgment: no information |
| Incomplete outcome data (attrition bias) Penile rigidity | Unclear risk | Judgment: 30/36 (83.3%) randomized participants in experimental group and 28/34 (82.4%) in control group were included in the analysis |
| Selective reporting (reporting bias) | Unclear risk | Judgment: the protocol was not found |
| Other bias | Low risk | Judgment: not found |

**Footnotes**

**CI:**confidence interval; **DM:** diabetes mellitus; **ED:** erectile dysfunction;**EDITS:** Erectile Dysfunction Inventory of Treatment Satisfaction; **EDV:** end diastolic velocity; **EHS:**Erection Hardness Score; **FMD:** flow mediated dilatation;**GAQ:** Global Assessment Questionnaire; **Hz:** hertz; **IAD:**intra-arterial diameter;**IIEF:**International Index of Erectile Function; **IIEF-5:** International Index of Erectile Function - 5 question; **IQR:** Interquartile Range; **LI-ESWT:**low intensity extracorporeal shock wave therapy; **MCID:** minimally clinically important difference; **mj/mm^2^:** millijoules per square millimeter; **NA:**not available; **NR:** not reported; **PDE5-I:** phosphodiesterase type 5 inhibitor; **PFE:** Pelvic Floor Exercise;**PSV:**peak systolic velocity; **QoL:** Quality of Life; **RI:** resistance index; **SD:**standard deviation; **SEP:**Sexual Encounter Profile; **SEPQ:** sexual encounter profile questionnaire; **SHIM:**Sexual Health Inventory for Men

**References to studies**

**Chung 2022 {published data only}**

- Chung E, Bailey W, Wang J. A prospective, randomized, double-blinded, clinical trial using a second-generation duolith SD1 low-intensity shockwave machine in males with vascular erectile dysfunction. World Journal of Men's Health 2022;41(1):94-100. [DOI: [10.5534/wjmh.210123](https://doi.org/10.5534/wjmh.210123)]

**Fojecki 2017 {published data only}**

- Fojecki GL, Tiessen S, Osther PJ. Effect of low-energy linear shockwave therapy on erectile dysfunction-a double-blinded, sham-controlled, randomized clinical trial. Journal of Sexual Medicine 2017;14(1):106-12. [DOI: [10.1016/j.jsxm.2016.11.307](https://doi.org/10.1016/j.jsxm.2016.11.307)]

**Harish 2017 {published data only}**

- Harish-Kumar G, Ravichandran R, Venugopal K. Low intensity extracorporeal shockwave therapy for erectile dysfunction: our experience. Indian Journal of Urology 2017;33(Suppl 1):S43–S178. [PMID: [PMC5307814](https://www.ncbi.nlm.nih.gov/pubmed/PMC5307814)]

**Kalyvianakis 2017 {published data only}**

- Kalyvianakis D, Hatzichristou D. Low-intensity shockwave therapy improves hemodynamic parameters in patients with vasculogenic erectile dysfunction: a triplex ultrasonography-based sham-controlled trial. Journal of Sexual Medicine 2017;14(7):891-7. [DOI: [10.1016/j.jsxm.2017.05.012](https://doi.org/10.1016/j.jsxm.2017.05.012)]

**Kalyvianakis 2022 {published data only}**

- Kalyvianakis D, Mykoniatis I, Pyrgidis N, Kapoteli P, Zilotis F, Fournaraki A, et al. The effect of low-intensity shock wave therapy on moderate erectile dysfunction: a double-blind, randomized, sham-controlled clinical trial. Journal of Urology 2022;208(2):388-95. [DOI: [10.1097/JU.0000000000002684](https://doi.org/10.1097/JU.0000000000002684)]

**Kennady 2023 {published data only}**

- Kennady EH, Bryk DJ, Ali MM, Ratcliffe SJ, Mallawaarachchi IV, Ostad BJ, et al. Low-intensity shockwave therapy improves baseline erectile function: a randomized sham-controlled crossover trial. Sexual Medicine 2023;11(5):qfad053. [DOI: [10.1093/sexmed/qfad053](https://doi.org/10.1093/sexmed/qfad053)]

**Kim 2020 {published data only}**

- Kim KS, Jeong HC, Choi SW, Choi YS, Cho HJ, Ha US, et al. Electromagnetic low-intensity extracorporeal shock wave therapy in patients with erectile dysfunction: a sham-controlled, double-blind, randomized prospective study. World Journal of Men's Health 2020;38(2):236-42. [DOI: [10.5534/wjmh.190130](https://doi.org/10.5534/wjmh.190130)]

**Kitrey 2016 {published data only}**

- Kitrey ND, Gruenwald I, Appel B, Shechter A, Massarwa O, Vardi Y. Penile low intensity shock wave treatment is able to shift PDE5i nonresponders to responders: a double-blind, sham controlled study. Journal of Urology 2016;195(5):1550-5. [DOI: [10.1016/j.juro.2015.12.049](https://doi.org/10.1016/j.juro.2015.12.049)]

**Olsen 2015 {published data only}**

- Olsen AB, Persiani M, Boie S, Hanna M, Lund L. Can low-intensity extracorporeal shockwave therapy improve erectile dysfunction? A prospective, randomized, double-blind, placebo-controlled study. Scandinavian Journal of Urology 2015;49(4):329-33. [DOI: [10.3109/21681805.2014.984326](https://doi.org/10.3109/21681805.2014.984326)]

**Ong 2022 {published data only}**

- Ong WL, Lechmiannandan S, Lim YL, Manoharan D, Lee SB. Early outcomes of short-course low intensity shockwave therapy (LiSWT) for erectile dysfunction: a prospective, randomized, double-blinded, sham-controlled study in Malaysia. Andrologia 2022;54(9):e14518. [DOI: [10.1111/and.14518](https://doi.org/10.1111/and.14518)]

**Ortac 2021 {published data only}**

- Ortac M, Ozmez A, Cilesiz NC, Demirelli E, Kadioglu A. The impact of extracorporeal shock wave therapy for the treatment of young patients with vasculogenic mild erectile dysfunction: a prospective randomized single-blind, sham controlled study. Andrology 2021;9(5):1571-8. [DOI: [10.1111/andr.13007](https://doi.org/10.1111/andr.13007)]

**Shendy 2021 {published data only}**

- Shendy WS, Elsoghier OM, El Semary MM, Ahmed AA, Ali AF, Saber-Khalaf M. Effect of low-intensity extracorporeal shock wave therapy on diabetic erectile dysfunction: randomised control trial. Andrologia 2021;53(4):e13997. [DOI: [10.1111/and.13997](https://doi.org/10.1111/and.13997)]

**Spivak 2018 {published data only}**

- Spivak L, Vinarov A, Platonova D, Demidko Y. Extracorporeal shock-wave therapy (ESWT) in treatment of ED: results of sham-controlled study. Journal of Urology 2018;199(4):e1175. [DOI: [10.1016/j.juro.2018.02.2876](https://doi.org/10.1016/j.juro.2018.02.2876)]

**Sramkova 2020 {published data only}**

- Sramkova T, Motil I, Jarkovsky J, Sramkova K. Erectile dysfunction treatment using focused linear low-intensity extracorporeal shockwaves: single-blind, sham-controlled, randomized clinical trial. Urologia Internationalis 2020;104(5-6):417-24. [DOI: [10.1159/000504788](https://doi.org/10.1159/000504788)]

**Srini 2015 {published data only}**

- Srini VS, Reddy RK, Shultz T, Denes B. Low intensity extracorporeal shockwave therapy for erectile dysfunction: a study in an Indian population. Canadian Journal of Urology 2015;22(1):7614-22. [PMID: [25694008](https://www.ncbi.nlm.nih.gov/pubmed/25694008)]

**Vardi 2012 {published data only}**

- Vardi Y, Appel B, Kilchevsky A, Gruenwald I. Does low intensity extracorporeal shock wave therapy have a physiological effect on erectile function? Short-term results of a randomized, double-blind, sham controlled study. Journal of Urology 2012;187(5):1769-75. [DOI: [10.1016/j.juro.2011.12.117](https://doi.org/10.1016/j.juro.2011.12.117)]

**Vinay 2017 {published data only}**

- Vinay J, Moreno D, Vives A, Rajmil O, Ruiz-Castane E, Sanchez-Curbelo J. Electrohydraulic low-intensity shockwave therapy for PDE5i-refractory erectile dysfunction: a prospective, randomized, placebo-controlled study. Journal of Urology 2017;197(4S):E1218. [DOI: [10.1016/j.juro.2017.02.2841](https://doi.org/10.1016/j.juro.2017.02.2841)]

**Vinay 2021 {published data only}**

- Vinay J, Moreno D, Rajmil O, Ruiz-Castane E, Sanchez-Curbelo J. Penile low intensity shock wave treatment for PDE5I refractory erectile dysfunction: a randomized double-blind sham-controlled clinical trial. World Journal of Urology 2021;39(6):2217-22. [DOI: [10.1007/s00345-020-03373-y](https://doi.org/10.1007/s00345-020-03373-y)]

**Xin 2017 {published data only}**

- Xin ZC, Guan RL, Cui WS, Zhang XD, Tian L, Xie Y, et al. Safety and efficacy of low-intensity extracorporeal shockwave in the treatment of vasculogenic erectile dysfunction: a multicenter, double-blind, randomized sham-controlled clinical trial. Journal of Urology 2017;197(4):E1345-6. [DOI: [10.1016/j.juro.2017.02.3145](https://doi.org/10.1016/j.juro.2017.02.3145)]

**Yang 2019 {published data only}**

- Yang L, Chen X, He D. Linear focus low-intensity extracorporeal shockwave therapy in the treatment of erectile dysfunction: a multi-center, double-blinded, prospective, randomized, placebo-controlled study. European Urology Supplements 2019;18(1):e1624. [DOI: [10.1016/S1569-9056(19)31176-5](https://doi.org/10.1016/S1569-9056(19)31176-5)]

**Yee 2014 {published data only}**

- Yee C-H, Chan ES, Hou SS-M, Ng C-F. Extracorporeal shockwave therapy in the treatment of erectile dysfunction: a prospective, randomized, double-blinded, placebo controlled study. International Journal of Urology 2014;21(10):1041-5. [DOI: [10.1111/iju.12506](https://doi.org/10.1111/iju.12506)]
