# Supplementary material for: Low‐intensity shockwave therapy for erectile dysfunction: An abridged Cochrane review
Source: BJU Int. 2026 Mar 30;137(6):949–57. doi: 10.1111/bju.70236 (PMC13168926; doi:10.1111/bju.70236)
Supplement: Supplementary file 3 — Table S2. Characteristics of excluded studies. [file BJU-137-949-s001.docx]

**Table S2. Characteristics of excluded studies**

**Ergun O, Kim K, Kim MH, Hwang EC, Blair Y, Gudeloglu A, Parekattil S, Dahm P**
<https://doi.org/10.1002/14651858.CD013166.pub3>

*The material in this section has been supplied by the author(s) for publication under a Licence for Publication and the author(s) are solely responsible for the material. Cochrane has reviewed this material, but Cochrane has not copyedited, formatted or proofread. Cochrane accordingly gives no representations or warranties of any kind in relation to, and accepts no liability for any reliance on or use of, such material.*

**Characteristics of excluded studies**

**Table of contents**

- [Studies ordered by Study ID](https://www.cochranelibrary.com/cdsr/doi/10.1002/14651858.CD013166.pub3/supplementarymaterials/CD013166-SUP-03-characteristicsOfExcludedStudies.html#studies)
  - [Cohen 2015](https://www.cochranelibrary.com/cdsr/doi/10.1002/14651858.CD013166.pub3/supplementarymaterials/CD013166-SUP-03-characteristicsOfExcludedStudies.html#id445962071400507884)
  - [Hattat 2012](https://www.cochranelibrary.com/cdsr/doi/10.1002/14651858.CD013166.pub3/supplementarymaterials/CD013166-SUP-03-characteristicsOfExcludedStudies.html#id445962071400507890)
  - [Kałka 2020](https://www.cochranelibrary.com/cdsr/doi/10.1002/14651858.CD013166.pub3/supplementarymaterials/CD013166-SUP-03-characteristicsOfExcludedStudies.html#id445962071400507891)
  - [Ladegaard 2021](https://www.cochranelibrary.com/cdsr/doi/10.1002/14651858.CD013166.pub3/supplementarymaterials/CD013166-SUP-03-characteristicsOfExcludedStudies.html#id445962071400507886)
  - [Mykoniatis 2022](https://www.cochranelibrary.com/cdsr/doi/10.1002/14651858.CD013166.pub3/supplementarymaterials/CD013166-SUP-03-characteristicsOfExcludedStudies.html#id445962071400507885)
  - [Pokorny 2015](https://www.cochranelibrary.com/cdsr/doi/10.1002/14651858.CD013166.pub3/supplementarymaterials/CD013166-SUP-03-characteristicsOfExcludedStudies.html#id445962071400507887)
  - [Yamacake 2019](https://www.cochranelibrary.com/cdsr/doi/10.1002/14651858.CD013166.pub3/supplementarymaterials/CD013166-SUP-03-characteristicsOfExcludedStudies.html#id445962071400507889)
- [References to studies](https://www.cochranelibrary.com/cdsr/doi/10.1002/14651858.CD013166.pub3/supplementarymaterials/CD013166-SUP-03-characteristicsOfExcludedStudies.html#references)

**Studies ordered by Study ID**

| **Study** | **Reason for exclusion** |
| --- | --- |
| Cohen 2015 | Ineligible study design |
| Hattat 2012 | Ineligible study design |
| Kałka 2020 | Ineligible study design |
| Ladegaard 2021 | Ineligible patient population |
| Mykoniatis 2022 | Ineligible intervention |
| Pokorny 2015 | Ineligible study design |
| Yamacake 2019 | Ineligible patient population |

**References to studies**

**Cohen 2015 {published data only}**

- Cohen DJ. Comment on: Extracorporeal shockwave therapy in the treatment of erectile dysfunction: a prospective, randomized, double-blinded, placebo controlled study. CH Yee, ES Chan, SS Hou, CF Ng. Int J Urol 2014;21:1041-5. Sexual Medicine 2015;3(3):132-3.

**Hattat 2012 {published data only}**

- Hattat H, Hattat E, Hattat I. Patient and partner satisfaction from extracorporeal shockwave therapy (ESWT) for ED treatment. Journal of Sexual Medicine 2012;9:316. [DOI: [10.1111/jsm.12007-1](https://doi.org/10.1111/jsm.12007-1)]

**Kałka 2020 {published data only}**

- Kałka D, Gebala J, Biernikiewicz M. Re: electromagnetic low-intensity extracorporeal shock wave therapy in patients with erectile dysfunction: a sham-controlled, double-blind, randomized prospective study. European Urology 2020;78(1):110-1. [DOI: [10.1016/j.eururo.2020.02.018](https://doi.org/10.1016/j.eururo.2020.02.018)]

**Ladegaard 2021 {published data only}**

- Ladegaard PB, Mortensen J, Skov-Jeppesen SM, Lund L. Erectile dysfunction a prospective randomized placebo-controlled study evaluating the effect of low-intensity extracorporeal shockwave therapy (LI-ESWT) in men with erectile dysfunction following radical prostatectomy. Sexual Medicine 2021;9(3):100338. [DOI: [10.1016/j.esxm.2021.100338](https://doi.org/10.1016/j.esxm.2021.100338)]

**Mykoniatis 2022 {published data only}**

- Mykoniatis I, Pyrgidis N, Zilotis F, Kapoteli P, Fournaraki A, Kalyvianakis D, et al. The effect of combination treatment with low-intensity shockwave therapy and tadalafil on mild and mild-to-moderate erectile dysfunction: a double-blind, randomized, placebo-controlled clinical trial. Journal of Sexual Medicine 2022;19(1):106-15. [DOI: [10.1016/j.jsxm.2021.10.007](https://doi.org/10.1016/j.jsxm.2021.10.007)]

**Pokorny 2015 {published data only}**

- Pokorny P, Turcan P, Prochazka M, Prochazkova J. Evaluation of radial extracorporeal shock wave therapy in the treatment of erectile dysfunction. Journal of Sexual Medicine 2015;12(S3):233. [DOI: [10.1111/jsm.12872_2](https://doi.org/10.1111/jsm.12872_2)]

**Yamacake 2019 {published data only}**

- *Yamacake K, Carneiro F, Cury J, Lourenco R, Francolin PC, Piovesan AC, et al. Low-intensity shockwave therapy for erectile dysfunction in kidney transplant recipients. A prospective, randomized, double blinded, sham-controlled study with evaluation by penile Doppler ultrasonography. International Journal of Impotence Research 2019;31(3):195-203. [DOI: [10.1038/s41443-018-0062-2](https://doi.org/10.1038/s41443-018-0062-2)]
- NCT02412345. Extracorporeal shockwave therapy in the treatment for erectile dysfunction in male renal transplant recipients. clinicaltrials.gov/study/NCT02412345 (first posted 9 April 2015).
- Yamacake K, Carneiro F, Lourenco R, Piovesan AC, Srougi M, Nahas WC, et al. Low-intensity extracorporeal shockwave therapy improves erectile dysfunction in kidney transplant recipients. Results of a prospective, randomized, double blinded, sham controlled study. Journal of Urology 2019;201(S4):E385. [DOI: [10.1097/01.JU.0000555751.59219.9d](https://doi.org/10.1097/01.JU.0000555751.59219.9d)]
